# Medicinal plants utilized in the management of epilepsy in Ethiopia: ethnobotany, pharmacology and phytochemistry

**DOI:** 10.1186/s13020-022-00686-5

**Published:** 2022-11-19

**Authors:** Yihenew Simegniew Birhan

**Affiliations:** Department of Chemistry, College of Natural and Computational Sciences, Debre Markos University, P.O. Box 269, Debre Markos, Ethiopia

**Keywords:** Epilepsy, Medicinal plants, Anticonvulsant activity, Antiepileptic activity, Ethiopia

## Abstract

Epilepsy is a common central nervous system (CNS) disorder that affects 50 million people worldwide. Patients with status epilepticus (SE) suffer from devastating comorbidities and a high incidence of mortalities. Antiepileptic drugs (AEDs) are the mainstream treatment options for the symptomatic relief of epilepsy. The incidence of refractory epilepsy and the dose-dependent neurotoxicity of AEDs such as fatigue, cognitive impairment, dizziness, attention-deficit behavior, and other side effects are the major bottlenecks in epilepsy treatment. In low- and middle-income countries (LMICs), epilepsy patients failed to adhere to the AEDs regimens and consider other options such as complementary and alternative medicines (CAMs) to relieve pain due to status epilepticus (SE). Plant-based CAMs are widely employed for the treatment of epilepsy across the globe including Ethiopia. The current review documented around 96 plant species (PS) that are often used for the treatment of epilepsy in Ethiopia. It also described the in vivo anticonvulsant activities and toxicity profiles of the antiepileptic medicinal plants (MPs). Moreover, the phytochemical constituents of MPs with profound anticonvulsant effects were also assessed. The result reiterated that a lot has to be done to show the association between herbal-based epilepsy treatment and in vivo pharmacological activities of MPs regarding their mechanism of action (MOA), toxicity profiles, and bioactive constituents so that they can advance into the clinics and serve as a treatment option for epilepsy.

## Introduction

Epilepsy is a common central nervous system (CNS) disorder and the fourth-largest cause of disease burden worldwide [1]. It is mainly characterized by recurrent, unprovoked seizures, which may trigger anxiety, depression, cognitive decline, schizophrenia, autism that can deteriorate the quality of life (QOL) and increase the incidence of mortality in patients [2, 3]. An imbalance instigated by inhibition of the excitatory γ-aminobutyric acid (GABA)-mediated neurotransmission and activation of inhibitory glutamatergic neurotransmission within the brain including hippocampal, neocortical, cortico-thalamic, and basal ganglia network is often implicated in the pathogenesis of epileptic seizures (ES) [4]. Epilepsy can emanate from a genetic predisposition of the brain to generate seizures or may be caused by brain damage due to tumor, injury, stroke, infection, etc. [5] that can elicit a wide array of abnormalities resulting in seizure generation [6]. According to WHO 2019 factsheet, approximately 50 million people around the globe are suffering from unpleasant symptoms and comorbidities resulting from ES [7]. It is reported that almost 80% of epilepsy cases are found in low—and middle-income countries (LMICs) [4] due to lack of sufficient antiepileptic drugs (AEDs), high cost if any AEDs available, and undesirable outcomes of the existing AEDs [8]. In the case of Ethiopia, epilepsy is one of the 20 leading causes of mortality, and 5.2 out of 1000 people are prone to ES in their lifetime [9, 10]. In general, epilepsy has substantial economic implications, predominantly in Africa, as it triggered a great burden on the underprivileged healthcare system of poor nations [11] as well as on patients owing to the epilepsy-bound poor QOL, stigma, and discrimination in patients and relatives [12] that could ominously increase healthcare expenditure and diminish overall productivity [10].

Modulating the activity of GABAergic, glutamatergic, purinergic neurotransmissions, cholinergic pathways and ATPases is a viable option for the treatment of epilepsy [13]. Attempts have been made to exploit the aforementioned neurotransmission pathways and enzymes implicated in epileptogenesis for the design of novel chemical agents to ameliorate the neurological deficits responsible for the progression of epilepsy. Thus far, more than 30 AEDs have been approved for clinical use [14]. However, the AEDs succeeded only in the symptomatic relief of epilepsy in patients without significantly correcting the underlying biochemical aberrations involved in epileptogenesis [15]. Currently, the treatment of epilepsy has mainly relied on such AEDs which can make patients free of seizures upon proper treatments regimens. Although the existing AEDs are effective in the suppression of seizures in the vast majority of epilepsy patients, 30% of them (15% of children and 34% adults) developed resistance towards AEDs, consequently, nonresponsive towards AEDs [16, 17]. Moreover, the dose-dependent neurotoxicity of AEDs such as fatigue, cognitive impairment, dizziness, attention-deficit behavior, and other side effects are the major bottlenecks in epilepsy treatment [8]. Patients with refractory ES are at increased risk of mortality and morbidity. Adjuvant therapies and AEDs along with ketogenic diet supplements are employed for the treatment of refractory ES [17]. Patients with untreated and/or refractory epilepsy are often desperate to seek nonconventional treatments including but not limited to complementary and alternative medicines (CAMs) [18]. The unaffordable price of newer AEDs and the wider treatment gaps have inspired researchers to focus on plants in the search for safe and effective drugs for the treatment of ES.

### Current trends in the treatment of epileptic seizures

AEDs are pretty effective in the treatment of epilepsy if patients properly comply with treatment regimens. However, they are overpriced and seldom possess devastating and inevitable side effects resulting in poor patient compliance [19]. Treatment compliance or adherence is a major factor that can dictate the outcomes of AEDs in controlling the incidence of seizure attacks [20]. There is ample evidence suggesting the presence of a huge treatment gap among epilepsy patients in LMICs ranging from 25 to 100% [21]. In Africa, epilepsy is associated with fear, misunderstanding, witchcraft, discrimination and social stigmatization of patients and their families that can be considered as a driving force for the observed huge treatment gaps due to failure in several intervention mechanisms employed and persistent antiepileptic medications non-adherences (AEMNAs) [22]. Epileptic patients experiencing AEMNAs are more prone to have suboptimal treatment outcomes, recurrent seizure attacks, intermittent hospital admissions, increased healthcare expenditure, lowered level of productivity, and thereby deteriorated QOL [23]. For instance, in Ethiopia, the prevalence of AEMNAs was found to be in the range of 21.8–68%. Poor healthcare system and medical services, lack of medication access, economic constraints, antiepileptic medication side effects, and poor seizure control status are among the factors which significantly contributed to the high burden of AEMNAs in Ethiopia [24]. Moreover, the association of epilepsy with spiritual and predestined fate as well as the presence of different cultural and spiritual beliefs with potential impacts to enforce people to prefer CAMs for the treatment of “spiritual disease” such as epilepsy [25] have significantly contributed to the high incidence of AEMNAs in different parts of Ethiopia. Overall, AEMNAs resulted in treatment failure which in turn triggered devastating social consequences, life-threatening comorbidities, employment restriction, physical injuries, and increased mortality [23]. For instance, in sub-Saharan Africa, untreated ES are the common causes of death with status epilepticus (SE), drowning, falls, burns, and sudden death contributing to epilepsy-associated mortality [26]. A study conducted on 119 patients in Ethiopia revealed that about 58% of epileptic patients who acquired generalized tonic–clonic seizures (GTCS) at a baseline evaluation with a frequency of ≤ 8 times, 23.3% of them died [27]. Another study revealed that among 316 persons with epilepsy, 20 (6.3%) died within 2 year period mostly due to SE and burn [28]. Accordingly, improving the patient compliance towards the existing AEDs through novel intervention approaches and bringing CAMs, especially antiepileptic herbal formulation, into modern pharmacy shelves is an option in the long term to tackle seizure-related morbidity and mortality.

### Importance of complementary and alternative medicine in Ethiopia

According to National Center for Complementary and Alternative Medicines (NCAM), CAMs are defined as a traditional healthcare system comprised of biological, spiritual, alternative, physical, and energy therapies [18]. A biological form of CAM that depends on natural products is commonly sought for the treatment of different diseases worldwide [29]. It uses medicinal herbs, medicinal animals, dietary supplements, antioxidants, minerals, vitamins, etc. alone or in combination to diagnose, prevent and treat different ailments [30]. Traditional medicines (TMs) of plant origin have become an integral part of the healthcare system of developed and developing countries [31] where 60% of the population entirely depend on them to relieve different types of ailments. Medicinal plants (MPs) have played a vital role in the treatment of human and livestock ailments since immemorial [32] partly due to the presence of bioactive secondary metabolites. Africa is the home of massive biodiversity rich in different types of animals and PS. The continent is likely to have approximately 45,000 PS of which 5000 species have medicinal importance [33]. Ethiopia is among the most diverse country located in East Africa containing approximately 6500–7000 PS (12% of them are endemic) in its flora [34]. It is also endowed with several languages, diverse cultures, and beliefs which are the driving force for the existence of traditional medical system plurality in the country [35]. Ethiopians have been using MPs and medicinal animals for the prevention, diagnosis, and treatment of different ailments since immemorial [36–40]. The healthcare demand of 80% of the people and 90% livestock in the country largely hinged on different PS [35]. Nearly 800 MPs are constantly employed to treat around 300 physical and mental diseases in the traditional healthcare system of Ethiopia [41]. The economic implication of MPs is noteworthy in Ethiopia. It is estimated that approximately 56,000 tons of wild MPs were collected per annum, which can potentially inject two billion Birr into the economy [42]. Such magnitude of MPs consumption is strongly associated with the accessibility, economic affordability, and cultural acceptability of MPs in different communities of Ethiopia [43].

### Data sources and search strategy

The present review describes the ethnobotany of MPs used to treat epilepsy and related symptoms in Ethiopia. It also focuses on the in vivo experimental evidence about the pharmacological efficacy of MPs in attenuating seizures in different animal models and on the type of bioactive compounds with profound anticonvulsant outcomes from the phytochemical investigation of MPs to establish a solid foundation for future research to develop plant-based antiepileptic agents. For this purpose, ethnobotanical data about the antiepileptic MPs found in Ethiopia were searched and downloaded from online research databases (PubMed, Medline, Web of Science, Google Scholar, Science Direct, and other institutional repositories) written in English using specific keywords such as “medicinal plants”, “medicinal herbs”, “ethnobotanical study”, “traditional medicine”, “traditional medication”, “plant remedies”, “herbal remedies”, “traditional healers”, “indigenous knowledge”, “folk medicine”, “traditional healers” + “Ethiopia”. Plant use reports for epilepsy and related symptoms were compiled and examined in terms of the habit of the MPs, parts used, condition of remedy preparation, route of administration, number of use citation (by Districts), target groups, etc. Based on the ethnobotanical information, a combination of keywords such as “scientific name of MPs” + “convulsions”, “anticonvulsant”, “seizure”, “antiseizure”, “epilepsy”, “antiepileptic”, “epileptic seizure”, “phytochemical investigation”, “active compounds”, “phytochemical screening”, “phytoconstituents”, “secondary metabolites”, “toxicity profiles”, etc. were used to search and collect relevant data on MPs with in vivo antiepileptic activities, toxicity profiles and to identify the phytochemicals (with already known anticonvulsant activities) present in the target MPs. The in vivo antiepileptic activities of MPs were analyzed based on the type of seizure-inducing agents, animal model, effective doses, and observed outcomes.

## Results and discussion

### Ethnobotany of medicinal plants used for the treatment of epilepsy

#### Plant distribution across families and geography

In this review, a total of 96 PS was found to have traditional healthcare prominence for the treatment of epilepsy and related symptoms in Ethiopia (Table 1). Of which 79 and 8 PS (*Agrocharis melanantha*, *Artemisia abyssinica*, *Crotalaria spinose, Cucurbita pepo*, *Erianthemum dregei*, *Myrica salicifolia*, *Solanum incanum*, and *Vigna membrancea*) were used to suppress ES in humans and animals, respectively. *Arundinaria alpina, Azadirachta indica*, *Croton macrostachyus*, *Echinops Kebericho*, *Embelia schimperi*, *Nicotiana tabacum*, *Ocimum lamiifolium*, *Satureja abyssinica* and *Vernonia amygdalina* were used to treat both human and livestock epilepsy cases. The reported MPs were distributed across 43 families and the highest occurrence belonged to Asteraceae (9, 20.93%), Fabaceae (8, 18.6%), Euphorbiaceae (7, 16.27%), Solanaceae (5, 11.63%), Lamiaceae (4, 9.3%) and Rubiacea (4, 9.3%). Apocynaceae, Celastraceae, and Rutaceae were represented by 3 (6.98%) PS each. In addition, Apiaceae, Cucurbitaceae, Verbenaceae, Malvaceae, Myrsinaceae, Myrtaceae, Oleaceae, Polygonaceae and Vitaceae families possessed 2 (4.65%) PS each. Other 26 families possessed a single PS effective against epilepsy in Ethiopia. Asteraceae, Fabaceae, Euphorbiaceae, and Solanaceae are the dominant families commonly found in the Ethiopian and Eritrean flora [44]. Thus, the mere presence of such PS in a relatively higher number in the antiepileptic MPs list is not a surprise. Overall, the data showed the cultural significance and medicinal importance of Asteraceae, Fabaceae, Euphorbiaceae, and Solanaceae families in the management of ES in Ethiopia. The dominance of Asteraceae, Fabaceae, Euphorbiaceae, and Solanaceae families were also reported in several ethnobotanical surveys conducted to document the MPs and associated indigenous knowledge used to treat different ailments in Ethiopia [45, 46].

**Table 1:** Ethnobotanical data of MPs used to treat epilepsy and related symptoms in Ethiopia

No.	Scientific name	Family	GF	PU	CP	ROA	TGs	Study areas	Refs.
1	*Acacia seyal* Delile	Fabaceae	T	B	D	N	Hu	Amaro District, SNNPR	[62]
2	*Acalypha fruticosa* Forssk	Euphorbiaceae	Sh	L	F	O	Hu	Yalo District, AfR	[63]
3	*Acokanthera schimperi* (A. DC.) Benth. & Hook.f. ex Schweinf	Apocynaceae	Sh	R	F/D	–	Hu	Enarso District, AR	[64]
4	*Agrocharis melanantha* Hochst	Apiaceae	H	R	F	N	Li	Bale Mountain National Park, OR	[65]
5	*Ajuga integrifolia*, Buch.-Hamn	Lamiaceae	H	L	D	O	Hu	Ghimbi District, Selale Mountain Ridges, Jimma Zone, OR	[53, 66, 67]
				Ap	F	O	Hu	Borecha District, OR	[68]
6	*Ampelocissus bombycina* (Baker) Planch	Vitaceae	Cl	R	F	O	Hu	Hawassa Zuria District, SNNPR	[59]
7	*Artemisia abyssinica* Sch. Bip. Ex A. Rich	Asteraceae	H	R	F	N	Li	Bale Mountain National Park, OR	[65]
8	*Artemisia afra* Jacq. Ex Willd	Asteraceae	H	L, R, SB	F	N	Hu	Bale Mountains National Park, OR	[69]
9	*Arundinaria alpina* K. Schum	Poaceae	T	L, Bu	F	O	Hu/Li	Dawuro Zone, SNNPR	[70]
10	*Asparagus africanus* Lam	Asparagaceae	Sh	L, R, SB	F/D	N	Hu	Ankober & Enarj Enawga Districts, AR	[71]
11	*Asplenium aethiopicum* (Kunth) mett	Aspleniaceae	H	L, R	F	N	Hu	Ankober District, AR	[71]
12	*Azadirachta indica* A. Juss	Meliaceae	T	L	F	O	Hu/Li	Adwa District, TR	[72]
13	*Balanites aegyptica* (L.) Del	Balantiaceae	T	R	–	N	Hu	Chifra District, AfR	[73]
14	*Biophytum umbraculum* Welw	Oxalidaceae	H	R	F	O	Hu	Dawuro Zone, SNNPR	[60]
15	*Brachiaria brizontha* (A. Rich.) Stapf	Poaceae	H	R	F	O	Hu	Dawuro Zone, SNNPR	[60]
16	*Brucea antidysenterica* J.F.Mill	Simaroubaceae	Sh	L	F	D	Hu	Adwa District, TR	[74]
17	*Breonadia salicina* (Vahl) Hepper & Wood	Rubiaceae	T	S	F/D	O	Hu	Berta Ethnic Group, BGR	[75]
18	*Buddleja polystachya*	Luganiaceae	T	L, R, B	D	O, N	Hu	Dawuro Zone, SNNPR	[60, 76]
19	*Calpurnia aurea* (Ait.) Benth	Fabaceae	Sh	R	F/D	O	Hu	Berta Ethnic Group, BGR	[75]
20	*Capparis tomentosa* Lam	Capparidaceae	Cl	R	D	N	Hu	Enarj Enawga District, AR; Asgede Tsimbila District, TR	[77, 78]
21	*Carissa edulis* (Forssk). Vahl	Apocynaceae	Sh	R	–	–	Hu	Asgede Tsimbila District, TR	[78]
22	*Caucanthus auriculatus* Forssk	Malpighiaceae	Cl	L	F	O	Hu	Gurage, Mareqo, Qebena, & Silti, SNNPR	[79]
23	*Caylusea abyssinica* (Fresen.) Fisch. & C.A.Mey	Resedaceae	H	L, R	F	O	Hu	Hamar District, SNNPR	[80]
24	*Chenopodium ambrosioides* L	Chenopodiaceae	H	L	F	O, N	Hu	Dawuro Zone, SNNPR	[70]
25	*Cissus petiolata* Hook. f	Vitaceae	Cl	S	–	D	Hu	Tahtay Koraro, Medebay Zana & Asgede Tsimbla, TR	[81]
26	*Celosia polystachia* (Forssk.) C.C. Towns	Amaranthaceae	H	L	F	O	Hu	Yalo District, AfR	[63]
27	*Clerodendrum myricoides* (Hochst.) R.Br. Ex Vatke	Verbenaceae	Sh	L	F	D	Hu	Bale Mountains National Park, OR; Asgede Tsimbila District, TR	[69, 78]
28	*Clutia abyssinica* Jaub	Euphorbiaceae	Sh	L	F	D	Hu	Aseko District, OR	[82]
29	*Crotalaria spinosa* Hochst. ex Benth	Fabaceae	H	L	F	O	Li	Mana Angetu District, OR	[83]
30	*Croton macrostachyus* Del	Euphorbiaceae	T	SB	F/D	O	Hu	Mana Angetu District, OR	[83]
				L	–	–	Hu	Asgede Tsimbila District, TR	[78]
				L	F/D	O	Li	Mana Angetu District, OR	[83]
31	*Cucumis ficifolius* A. Rich	Solanaceae	H	R, L	F	O	Hu	Asendabo District, OR	[84]
32	*Cucurbita pepo* L	Cucurbitaceae	Cl	L	F	O	Li	Mana Angetu District & Jimma Zone, OR	[67, 83]
33	*Desmodium repandum* (Vahl) DC	Fabaceae	H	R	F/D	N	Hu	Ankober District, AR	[71]
34	*Dicrocephula integrifolia* (L. f.) Kuntaze	Asteraceae	H	L	F	N, D	Hu	Dawuro Zone, SNNPR	[60, 76]
35	*Dregea schimperi* (Decne.) Bullock	Apocynaceae	Cl	L	F	O	Hu	Gurage, Mareqo, Qebena & Silti, SNNPR	[79]
36	*Echinops Kebericho* Mesfin	Asteraceae	H	R	F	N	Hu	Kembatta Tembaro Zone, SNNPR	[85]
				R	D	N	Li	Baso Liben & Debre Elias Districts, AR	[86]
				R, RB	F	O, N	Hu/Li	Dawuro Zone, SNNPR	[70]
37	*Embelia schimperi* Vatke	Myrsinaceae	T	Fr	F	O	Hu	Debark Woreda, AR	[87]
				R	D	O	Li	Baso Liben & Debre Elias Districts, AR	[86]
38	*Erianthemum dregei* (Eckl and Zeyh.) V. Tiegh	Loranthaceae	T	L, S, R	F/D	O	Li	Mana Angetu District, OR	[83]
39	*Eucalyptus globulus* Labull	Myrtaceae	T	L, Se	F/D	O, N	Hu	Kembatta Tembaro Zone, SNNPR	[85]
40	*Euphorbia tirucalli* L	Euphorbiaceae	Sh	R	F/D	O	Hu	Amaro District, SNNPR	[62]
41	*Fagaropsis angolensis* (Engl.) Milne-Redh	Rutaceae	T	Se, L	F	O	Hu	Kochere District, SNNPR	[88]
42	*Ficus vasta* Forssk	Moraceae	T	B	D	N, D	Hu	Dega Damot District, AR	[52]
43	*Galinirea coffeoides*	Rubiaceae	Sh	L, R	F	O	Hu	Dawuro Zone, SNNPR	[60, 76]
44	*Gloriosa superba* L	Colchicaceae	Sh	L	F	O	Hu	Harla & Dengego valleys, DDAC	[89]
				R	F/D	O	Hu	Mana Angetu District, OR	[83]
45	*Guizotia scabra* (Vis) Chiov	Compositae	H	R	D	O	Hu	Ada'a District, OR	[90]
46	*Hagenia abyssinica* (Bruce) J.F. Gmel	Rosaceae	T	Fl	–	–	Hu	Bale Rural Communities, OR	[91]
47	*Hypericum quartinianum* A. Rich	Hypericaceae	Sh	L	D	D	Hu	Around Fiche District, OR	[92]
48	*Indigofera articulata* Gouan	Fabaceae	Sh	L, R	F	O	Hu	Yalo District, AfR	[63]
49	*Indigofera coerulea* Roxb	Fabaceae	Sh	R	F	O	Hu	Jeldesa Cluster, DDAC	[93]
50	*Inula confertiflora* A. Rich	Asteraceae	Sh	L	F	N	Hu	Enarj Enawga District, AR	[77]
51	*Jatropha curcas* L	Euphorbiaceae	Sh	Se	F	O	Hu	Gurage, Mareqo, Qebena & Silti, SNNPR	[79]
52	*Jasminum abyssinicum* Hochst. Ex DC	Oleaceae	Cl	L	F	N	Hu	Kembatta Tembaro Zone, SNNPR	[85]
53	*Justitia schimperiana* Hochst. ex Nees	Acanthaceae	Sh	L	F	O, D	Hu	Dawuro Zone, SNNPR	[70]
54	*Lagenarin abyssinica* (Hoof. f) C. Jeffrey	Cucurbitaceae	H	L	F	N	Hu	Asendabo District, OR	[84]
55	*Laggera crispata* (Vahl) Hepper & Wood	Asteraceae	Sh	R	F	O	Hu	Yilmana Densa & Quarit Districts, AR	[42]
56	*Lobelia gibberoa* Hemsl	Lobeliaceae	T	Se	D	O	Hu	Gubalafto District, AR	[61]
57	*Maytenus gracilipes* (Welw.ex Oliv) Exell	Celastraceae	Sh	L	D	O	Hu	Bale Mountains National Park, OR	[69]
58	*Maytenus heterophylla* (Eckl. & Zeyh.) Robson	Celastraceae	Sh	L	F	O	Hu	Gurage, Mareqo, Qebena & Silti, SNNPR	[79]
59	*Maytenus senegalensis* (Lam.) Excell	Celastraceae	Sh	Se	F/D	O	Hu	Wonago District, SNNPR	[45]
60	*Myrica salicifolia* Hochst. ex A. Rich	Myrsinaceae	T	B	D	N	Li	Hulet Eju Enese District, AR	[35]
61	*Nicotiana tabacum* L	Solanaceae	Sh	R	D	O, N	Hu	Mana Angetu District, OR; Ankober District, AR	[71, 83]
				L	F	D, N	Hu	Fadis & Dugda Districts, OR; Ankober District, AR	[71, 94, 95]
				L	F	O	Li	Mana Angetu District, OR	[83]
62	*Ocimum canum* Sims	Lamiaceae	H	L	F	N	Hu	Dawuro Zone, SNNPR	[70]
63	*Ocimum lamiifolium* Hochst,ex Benth	Lamiaceae	H	L	F	O, N, D	Hu/Li	Dawuro Zone, SNNPR	[70]
64	*Olea europaea* L	Oleaceae	T	L	D	N	Hu	Hulet Eju Enese District, AR	[35]
65	*Olinia rochetiana* A. Juss	Oliniaceae	T	R	F/D	N	Hu	Ankober District, AR	[71]
66	*Opuntia ficus-indica (L.)* Miller	Cactaceae	H	L	F	D	Hu	Debark District, AR	[87]
67	*Pavetta abyssinica* Fresen	Rubiaceae	Sh	Bu, Se	F	N	Hu	Kembatta Tembaro Zone, SNNPR	[85]
68	*Pentas schimperiana* (A. Rich) Vatke	Rubiaceae	Sh	RB	F/D	O	Hu	Wonago District, SNNPR	[45]
69	*Plectranthus edulis* Vatke	Lamiaceae	H	L, R	-	O	Hu	Abay Chomen District, OR	[96]
70	*Pterolobium stellatum* Forsk. Brenan	Fabaceae	Sh	R	F/D	N	Hu	Hulet Eju Enese District, AR	[35]
				Wh	F	O	Hu	Bahir Dar Zuria District, AR	[97]
				L, R	F	N	Hu	Hamar District, SNNPR	[80]
71	*Rhamnus staddo* A. Rich	Rhamnaceae	Sh	L	F	N	Hu	Enarj Enawga District, AR	[77]
72	*Rhus vulgaris* Meikle	Anacardiaceae	Sh	L	F	O, N, D	Hu	Dawuro Zone, SNNPR	[70]
73	*Rumex nepajensis* Spreng	Polygonaceae	Sh	R	F	N	Hu	Borecha District, OR	[68]
74	*Ruta chalepensis* L	Rutaceae	Sh	L, Se	F	N	Hu	Hulet Eju Enese District, AR	[35]
75	*Satureja abyssinica* (Benth.) Briq	Lamiaceae	H	L	F	N	Hu/Li	Dawro Zone, SNNPR	[60, 76]
76	*Securidaca longepedunculata* Fres	Polygonaceae	T	R	D	N	Hu	Enemay District, AR	[39]
77	*Solanum incanum* L	Solanaceae	Sh	R	F	O	Li	Mana Angetu District, OR	[83]
78	*Sida rhombifolia* L	Malvaceae	H	R	–	N	Hu	Tahtay Koraro, Medebay Zana & Asgede Tsimbla, TR	[81]
79	*Sida schimperiana* Hochst. Ex A.Rich	Malvaceae	Sh	–	F	O	Hu	Wonago District, SNNPR	[45]
80	*Syzygium guineense* (Willd.) DC	Myrtaceae	T	S	D	O, N	Hu	Berta Ethnic Group, BGR	[75]
81	*Tragia cinerea* (Pax) Gilbert and Radcl.-Smith	Euphorbiaceae	Cl	R	D	O	Hu	Menz Gera-Midir District, AR	[98]
82	*Tynura pseudochina* L	Compositae	Sh	L	F	O	Hu	Borecha District, OR	[68]
83	*Urera hypselodendron* (Hochst.) ex A. Rich	Urticaceae	Cl	R	D	O	Hu	Hulet Eju Enese District, AR	[35]
84	*Vangueria volkensii* K.Schum	Rubiaceae	Sh	L, R	F	O	Hu	Hamar District, SNNPR	[80]
85	*Verbena bonariensis*	Verbenaceae	H	L	D	N	Hu	Mojana District, AR	[99]
86	*Vernonia amygdalina* Del	Asteraceae	Sh	L, B	F	O, D	Hu/Li	Dawuro Zone, SNNPR	[70]
87	*Vigna membrancea* (L.) A. Rich	Fabaceae	Cl	L, R	F/D	O	Li	Abay Chomen & Kersa Districts, OR	[55, 96]
88	*Withania somnifera* (L.) Dun	Solanaceae	Sh	R	F/D	O	Hu	Mana Angetu District, OR	[83]
89	*Xanthium stramonium* L	Solanaceae	H	L	F	D	Hu	Fadis District, OR	[95]
90	*Zingiber officinale* Roscoe	Zingiberaceae	H	R	F	O	Hu	Amaro District, SNNPR	[62]

*GF* growth forms, *T* Tree, *Sh* shrub, *H* herb and *Cl* climber, Plant *PU* parts used, *L* leaf (), *S* stem, *SB* stembark, *R* root, *RB* rootbark, *Bd* buds, *Ap* apex, *Se* seed, *Wh* whole plant, *Ar* aerial part (), *Bu* bulbs, *Lx* latex, *Fr* fruit, *Fl* flower and *Rh* rhizome, *CP* condition of preparation, *F* fresh, and *D* dry, *ROA* routes of administration, *O* Oral, *N* nasal, *D* dermal and *Au* auricular, *TGs* target groups, *Hu* Human and *Li* livestock, Reginal states of Ethiopia: *AR* amhara region, *AfR* Afar region, *BGR* Benshangul-Gumuz region, *DDAC* dire dawa administration council, *OR* oromia region, *TR* tigray region, *SNNPR* southern nations, nationalities and peoples and peoples region

**Fig. 1:**
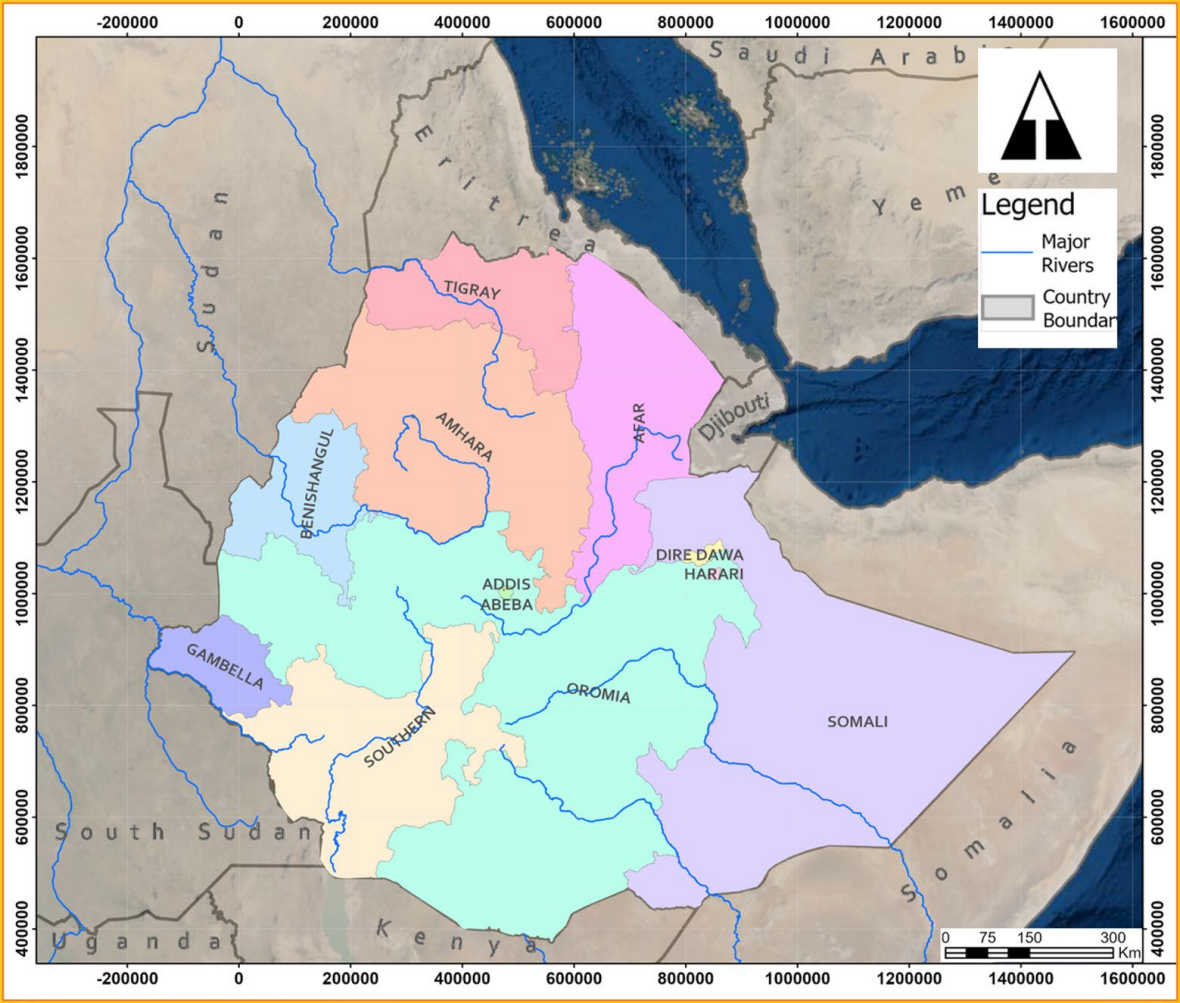
Location map of Ethiopia. The different colored areas represent the regional states in Ethiopia where the use of plant-based medicines are reported

TMs, especially MPs are routinely used for the management of different diseases in the traditional healthcare system of the Regional States of Ethiopia [47–53]. Although these Regional States share some common entities, they have distinct biodiversities, agro-ecology, cultures, livelihood, values, beliefs, etc. which nurture the indigenous knowledge and traditional practices of dwellers. Hence, multifaceted treatment approaches and miscellaneous traditional remedies are prevalent in different cultural groups of Ethiopia [36, 44, 54, 55]. In line with this fact, the present literature review reiterated that the use citations of antiepileptic MPs are widely distributed across the different regional states of Ethiopia (Fig. 1): Oromia (29 PS), Amhara (25 PS), Southern Nations, Nationalities and Peoples (33 PS), Afar (4 PS), Tigray (8 PS), Benshangul-Gumuz (3 PS) and Dire Dawa Administration Council (2 PS). More than 70% of MP species prescribed for the treatment of seizure in Ethiopia belonged to the three most populous and diverse regions, namely Oromia, Amhara, and the SNNP Regional States. This may be attributed to the presence of different biodiversities, cultural pluralities, and thereby rich indigenous MPs knowledge and practice in the regions. Despite the cross-cultural connections and neighborhood manifested by the long common border between Oromia and Amhara regions as well as Oromia and SNNP regions, the consensus of THs on antiepileptic MPs was quite low, only a few MPs were commonly used across the regions.

#### Parts used, condition of preparation, and mode of administration of MPs

Among the reported 95 MPs, shrubs accounted for 35 (36.46%) PS. Herbs 30 (31.25%) and trees 21 (21.88%) were the second and third most abundant growth forms of MPs. On the other hand, 10 (10.42%) MPs were climbers. The relative abundance of shrubs in Ethiopian flora and its accessibility in year-round may have contributed to higher use citation of shrubs in antiepileptic medication preparation [35]. The THs of Ethiopia preferred leaves, (66, 44. 59%) over other plant parts for the preparation of remedies. They also often used roots (52, 34.14%) and seeds (10, 6.76%) for the formulation of medicinal recipes. In addition, bulbs, stembark, rootbark, apex, rhizome, flowers, fruits, the whole plant, and aerial part of MPs were also used for the extraction of effective medicines for seizure. The presence of bioactive compounds, both in therapeutic abundance and variety, in leaves and roots may be associated with the curative effects of such recipes against epilepsy [56, 57]. Fresh organs of plants (81, 64.8%) were often employed for the preparation of antiepileptic medications in Ethiopia. Dry forms of plant parts (23, 18.4%) were also used for the preparation of remedies. Nearly 17% of plant parts were used regardless of the condition they exist (either fresh or dry). As fresh plant parts are rich in bioactive metabolites, they are frequently sought for the formulation of remedies not only for epilepsy but also for other ailments in Ethiopia. In addition, fresh plant parts are convenient to prepare medications using crushing, squeezing, maceration, infusion, decoction, etc., and can be ready for use in a short period as compared to dry plant organs [44].

Diverse approaches and strict procedures are followed by the THs for the preparation of remedies: abstraction of pharmacologically relevant crude extract or essential oils from different plant organs in Ethiopia [47, 58, 59]. Depending on the perceived knowledge of the THs, some may prefer crushing for remedy preparation while others may use tying or burning of the same plant part for the same ailment. The antiepileptic medications in Ethiopia were most commonly prepared using crushing, squeezing, maceration, pounding, grinding, decoction, etc. techniques. Water was the main extraction solvent employed in most preparations to tailor the concentration of the recipe to the supposed level of therapeutic efficacy and to avoid dose-related toxicities in patients [45]. Additives such as milk, “*tella*” (local beer), “*teff injera*” (flat bread), sugar, etc. [60–62] were used to improve the taste of the recipe and to enhance patient compliance towards the formulations. Most of the antiepileptic herbal formulations were administered through the oral route (63, 51.64%) by drinking, chewing, etc. followed by nasal (41, 33.61%) in the form of sniffing, smoking, and fumigation. Dermal route of administration (ROA) (18, 14.75%) (through fumigation and washing) was seldom employed for the delivery of antiseizure herbal medications in the Ethiopian context. Oral is described as the primary ROA in several ethnobotanical studies conducted elsewhere [48, 59] due to the fast onset of action and ease of application.

### Multiple medicinal plants prescriptions for the treatment of epilepsy

Combinations of two or more PS are seldom used to formulate remedies for epilepsy and related symptoms in Ethiopia and elsewhere [100]. This is based on the fact that the consumption of multiple MPs could have potential synergistic outcomes and thereby enhanced pharmacological activities. For instance, the roots of four MPs including *Guizotia scabra*, *Ajuga integrifolia*, *Foeniculum vulgare,* and *Withania somnifera* have been used for the preparation of remedy that can be taken through the oral route in Adaꞌa District, Oromia Regional State, Ethiopia [90] that can potentially attenuate convulsions in humans (Table 2). On the other hand, leaves of *Artemisia abyssinica*, *Brucea antidysentrica,* and *Cucumis ficifolius* were employed for the preparation of recipes effective against epilepsy, when taken orally, around Jimma, Oromia Regional State, Ethiopia [101]. Similarly, the leaves of *Nicotiana tabacum*, *Ocimum lamiifolium,* and *Withania somnifera* were also used for the preparation of remedies that can be applied externally (dermal route) to relieve seizure [102]. Herbalists living around Fiche District, Oromia Regional State, Ethiopia prepare a remedy for epilepsy from leaves of *Hypericum quartinianum*, *Podocarpus falactus*, and *Teclea nobilis* for external application through the nasal ROA [92]. The different classes of phytochemicals such as alkaloids, flavonoids, terpenoids, etc. present in these MPs and their combined effect in enhancing the relative abundance/concentration and amplifying the pharmacological efficacy through synergism may be associated with the preparation of efficient antiepileptic recipes from multiple MPs. *Ocimum lamiifolium*, *Nicotiana tabacum, Ruta chalepensis* and *Withania somnifera* were most frequently sought MPs for the preparation of antiseizure medications, each become part of two different formulations [35, 90, 94, 102]. The wide application of *Ocimum lamiifolium*, *Nicotiana tabacum, Ruta chalepensis* and *Withania somnifera* in different formulations might be due to the presence of convulsion-suppressive bioactive compounds in such MPs. For obvious reasons, the use of formulations of multiple MPs is a common practice in the treatment of epilepsy in different parts of the world [103].

**Table 2:** Ethnobotanical data of multiple MPs prescriptions used to treat epilepsy and related symptoms in Ethiopia

No.	Scientific name	Family	GF	PU	CP	ROA	Study area	Refs.
1	*Artemisia abyssinica* Sch. Bip. Ex A. Rich	Asteraceae	H	L	F	O	Jimma Area District, OR	[101]
2	*Brucea antidysentrica* J.F. Mill	Simaroubaceae	Sh	L	F			
3	*Cucumis ficifolius* A. Rich	Solanaceae	Cl	L	F			
1	*Embelia schimperi* Vatke	Myrsinaceae	T	Fr	F	O	Debark District, AR	[87]
2	*Guizotia abyssinica* (L. f.) Cass	Asteraceae	H	Se	D			
1	*Fagaropsis angolensis* (Engl.) Milne-Redh	Rutaceae	T	Se	D	O	Kochere District, SNNPR	[88]
2	*Solanum spp.*	Solanaceae	H	L	F			
1	*Guizotia scabra* (Vis) Chiov	Compositae	H	R	D	O	Ada'a District, OR	[90]
2	*Ajuga integrifolia*, Buch.-Hamn	Lamiaceae	H	R	F/D			
3	*Foeniculum vulgare* Mill	Apiaceae	H	R	F/D			
4	*Withania somnifera* (L.) Dun	Solanaceae	Sh	R	F/D			
1	*Hypericum quartinianum* A. Rich	Hypericaceae	Sh	L	D	D	Around Fiche District, OR	[92]
2	*Podocarpus falactus* (Thunb.) R. B. ex Mirb	–	T	L	F			
3	*Teclea nobilis* Del	Rutaceae	T	L	F			
1	*Nicotiana tabacum* L	Solanaceae	H	L	F	D	Dugda District, OR	[94]
2	*Ocimum lamiifolium* Hochst	Lamiaceae	H	L	F			
1	*Nicotiana tabacum* L	Solanaceae	H	L	F	D	Seru District, OR	[102]
2	*Ocimum lamiifolium* Hochst	Lamiaceae	H	L	F			
3	*Withania somnifera* (L.) Dun	Solanacae	Sh	L	F			
1	*Pterolobium stellatum* Forsk. Brenan	Fabaceae	Cl	R	F	N	Hulet Eju Enese District, AR	[35]
2	*Ruta chalepensis* L	Rutaceae	Sh	R	F			
1	*Ruta chalepensis* L	Rutaceae	Sh	L, Se	F	N	Hulet Eju Enese District, AR	[35]
2	*Allium sativum* L	Alliaceae	H	Bu	F/D			

*GF* growth forms, *T* Tree, *Sh* shrub, *H* herb, *Cl* climber, *PU* plant parts used, *L* leaf, *R* root, *Se* seed, *Ar* Aerial part, *Bu* bulbs and *Fr* fruit, *CP* condition of preparation, *F* Fresh and *D* dry, *ROA* routes of administration, *O* Oral, *N* nasal and *D* dermal, Reginal states of Ethiopia *AR* amhara region, *AfR* afar region, *BGR* benshangul-gumuz region, *DDAC* dire dawa administration council, *OR* Oromia region, *TR* Tigray region, *SNNPR* southern nations, nationalities and peoples and peoples region

### Global importance of the medicinal plants in the treatment of Epilepsy

Among the reported MPs for the treatment of epilepsy and related symptoms in Ethiopia, 34 PS were also routinely used for the same indications in different parts of the world including Africa, Asia, the Middle East, and Latin America (Table 3). Among these, *Carissa edulis* was the most popular (cited in six countries) antiepileptic MP frequently used to control seizure in Ethiopia, Nigeria, South Africa, Uganda, Malawi, and Kenya [104–108]. Similarly, *Maytenus senegalensis* was another well-known (cited in five countries) anticonvulsant MP in Africa including Ethiopia, Uganda, Zimbabwe, South Africa, and Guinea-Bissau [100, 106, 107, 109]. *Withania somnifera* was another multipurpose MP (cited in four countries) used to control convulsions in Ethiopia, Lesotho, India, and in East African countries [107, 110, 111]. Moreover, *Acacia seyal*, *Acalypha fruticosa*, *Allium sativum*, *Balanites aegyptica*, *Biophytum umbraculum*, *Clerodendrum myricoides*, *Euphorbia tirucalli*, *Indigofera arrecta*, *Maytenus heterophylla*, *Nicotiana tabacum,* and *Ruta chalepensis* were the other MPs reported for their usefulness against convulsions in at least three countries [100, 103, 106, 107, 112–126]. The remaining MPs: *Artemisia afra, Asparagus africanus*, *Azadirachta indica*, *Capparis tomentosa*, *Clutia abyssinica*, *Croton macrostachyus*, *Cucurbita pepo*, *Eucalyptus globulus*, *Indigofera articulata*, *Indigofera coerulea, Jatropha curcas*, *Myrica salicifolia*, *Olea europaea*, *Opuntia ficus-indica*, *Sida rhombifolia*, *Xanthium stramonium,* and *Zingiber officinale* were indicated for epilepsy in Ethiopia and at least one other country [105, 106, 108, 109, 118, 127–137]. The extensive use of MPs across different countries of the globe echoed the existence of shared ethnopharmacological knowledge among the THs, the importance of such MPs in the healthcare system of LMIC, especially in tropical and southern Africa, and more importantly, the pharmacological efficacy of the MPs in the treatment of epilepsy and related symptoms.

**Table 3:** List of MPs plants used to treat epilepsy and related symptoms in other parts of the world

No.	Scientific name	Family	GF	PU	Country/region	Refs.
1	*Acacia seyal*	Fabaceae	T	R	Tanzania and Uganda	[106, 112]
2	*Acalypha fruticosa*	Euphorbiaceae	Sh	L, R	Tanzania and Kenya	[113, 114]
3	*Allium sativum*	Alliaceae	H	Bu	India and Cameron	[103, 115]
4	*Artemisia afra*	Asteraceae	H	L	South Africa	[105]
5	*Arundinaria alpina*	Poaceae	T	R	Uganda	[138]
6	*Asparagus africanus*	Asparagaceae	Sh	R	Cameron	[127]
7	*Azadirachta indica*	Meliaceae	T	L	India	[128]
8	*Balanites aegyptica*	Balantiaceae	T	L, B, R	Mali and Saudi Arabia	[116, 117]
9	*Biophytum umbraculum*	Oxalidaceae	H	L, Wh	Cameron and Uganda	[115, 118]
10	*Capparis tomentosa*	Capparidaceae	Cl	L	Uganda	[106]
11	*Carissa edulis*	Apocynaceae	Sh	L,R, RB, Fr	Nigeria, South Africa, Uganda, Malawi and Kenya	[104–108]
12	*Chenopodium ambrosioides*	Chenopodiaceae	H	L	Democratic Republic of Congo	[139]
13	*Clerodendrum myricoides*	Verbenaceae	Sh	L, R	South Africa and Kenya	[100, 119]
14	*Clutia abyssinica*	Euphorbiaceae	Sh	R	Rwanda	[129]
15	*Croton macrostachyus*	Euphorbiaceae	T	B	Cameron	[140]
16	*Cucurbita pepo*	Cucurbitaceae	Cl	–	Nigeria	[130]
17	*Eucalyptus globulus*	Myrtaceae	T	L, B	Kenya	[131]
18	*Euphorbia tirucalli*	Euphorbiaceae	S	Lx, Ar	Somalia and East Africa	[120, 121]
19	*Indigofera arrecta*	Fabaceae	Sh	L, R	South Africa and Nigeria	[100, 122]
20	*Indigofera articulata*	Fabaceae	Sh	Wh	India	[132]
21	*Indigofera coerulea*	Fabaceae	Sh	L	India	[133]
22	*Jatropha curcas*	Euphorbiaceae	Sh	L	Nigeria	[134]
23	*Maytenus heterophylla*	Celastraceae	Sh	R	East Africa	[107]
24	*Maytenus senegalensis*	Celastraceae	Sh	L, R,	Uganda, Zimbabwe, South Africa and Guinea-Bissau	[100, 106, 107, 109]
25	*Myrica salicifolia*	Myrsinaceae	T	B	Uganda	[118]
26	*Nicotiana tabacum*	Solanaceae	H	L	Nigeria and Cameron	[115, 123, 124]
27	*Olea europaea*	Oleaceae	T	B, R, Fr	Kenya	[108]
28	*Opuntia ficus-indica*	Cactaceae	H	Fl	India	[135]
29	*Ruta chalepensis*	Rutaceae	Sh	Ar	Morocco and Mexico	[125, 126]
30	*Sida rhombifolia*	Malvaceae	H	Wh	India	[136]
31	*Syzygium guineense*	Myrtaceae	T	SB	West Africa	[109]
32	*Withania somnifera*	Solanacae	H	S, R	Lesotho, East Africa and India	[107, 110, 111]
33	*Xanthium stramonium*	Solanaceae	H	Wh	India	[141]
34	*Zingiber officinale*	Zingiberaceae	H	Rh	Japan	[137]

*GF* growth forms, *T* Tree, *Sh* shrub, *H* herb, *Cl* climber, *PU* plant parts used, *L* Leaf, *S* stem, *SB* stembark, *R* root, *RB* rootbark, *Wh* whole plant, *Ar* Aerial part, *Bu* bulbs, *Lx* latex, *Fr* fruit, and *Rh* rhizome

## Pharmacological evidence of reported medicinal plants

### Animal models for screening of anticonvulsant or antiepileptic agents

The anticonvulsant or antiseizure activity of MPs claimed by THs for the management of epilepsy could be verified by using different in vitro and in vivo experiments. In 1937, electrically-induced convulsions in cats were used to check the bioactivity of phenytoin, the first modern AED [142]. Later, this initiative paved the way for the discovery of other seizure models responsible for the discovery of more safe and efficacious second-generation AEDs such as lamotrigine, levetiracetam, topiramate, lacosamide, pregabalin, etc. [143]. The ability of crude extracts or bioactive compounds to suppress different forms of seizures can be examined by animal models by artificially induced convulsions using maximal electroshock (MES) or drugs such as pentylenetetrazol (PTZ), picrotoxin (PIC), strychnine (STR), pilocarpine (PLC), isonicotinic hydrazide acid (INH), Kainic acid (KA), 4-aminophylline (AMP), bicuculline (BIC), etc. [144]. The similarity in the pattern of seizure triggered by different stimuli in animal models with humans, simplicity upon execution, quick response rate, and most importantly, predictive clinical outcomes in humans [145] make the in vivo seizure models trustworthy in epilepsy research. In general, MES acute seizure tests characterized by tonic extensions of forelimbs in and hind limbs followed by all limb clonus in mice/rat; subcutaneous PTZ acute seizure tests manifested by myoclonic jerks followed by unilateral forelimb and bilateral clonus, vibrissae twitching in mice/rats and a Kindled rodent model of chronic hyperexcitability characterized by unilateral and bilateral forelimbs clonus that progresses to rearing and falling in rats are the most common and “clinically validated” models for early evaluation of AEDs [142]. Albeit, the aforementioned acute seizure models failed to trace bioactive compounds effective against refractory or drug-resistant seizures. Thus, there had been a pressing need for the discovery of alternative seizure models which can embrace the deviations observed in “clinically validated” models. More recently, several non-mammalian seizure models consisting of fruit flies (*Drosophila melanogaster*), medicinal leeches (*Hirudo verbena*), planaria, roundworms (*Caenorhabditis elegans*), tadpoles (*Xenopus laevis*), zebrafish (*Danio rerio*), etc. were recognized for their versatility to assess the anticonvulsant activities of synthesized compounds or plant extracts [146, 147]. Of which, the zebrafish larvae were the most frequently used seizure model because of its high fertility rate and development, similar CNS organization with mammals which can be observed in translucent egg and embryo make it ideal to study CNS disorders provoked by external stimuli [148]. PTZ, KA, PLC and electrical stimulation are employed to induce convulsions in in the aforesaid non-mammalian seizure models [147].

### In vivo pharmacological activities of antiepileptic medicinal plants

CAMs, especially herbal remedies are extensively used for the treatment of epilepsy across the globe due to their desirable treatment outcomes and tolerable side effects [144]. Moreover, herbal therapies may yield a new horizon for treating patients seeking inexpensive treatments for untreated epilepsy and experiencing refractory seizures. Taking the popularity of the MPs prescribed for treatment and management of epilepsy in different cultural groups across the globe into account, preliminary in vitro and/or in vivo pharmacological evaluation of MPs and phytochemical isolation of bioactive compounds have been conducted to test the validity of the hypothesis made by THs found elsewhere. Researchers employed different animal models to quantify the extent of suppression of different forms of seizures induced via MES, PTZ, PIC. STR, PLC, NIH, and BIC by the crude extracts or solvent fractions of MPs claimed to have potential anticonvulsant activities. This section highlighted the in vivo anticonvulsant activity of MPs (Table 4) whereby ethnobotanical studies conducted in Ethiopia and other parts of the world reiterated their profound pharmacological activities against epilepsy and related symptoms.

**Table 4:** Plant crude extracts with in vivo antiepileptic/anticonvulsant activities

No.	Scientific name	PU	Extract	Seizure-inducing stimuli	Animal models	Doses (mg/kg)	Treatment outcomes	Refs.
1	*Acalypha fruticosa*	Ar	CH	PTZ, MES & INH	Adult Swiss albino mice (25–30 g)	30–300	Protected the mice from PTZ and MES-induced convulsions. Delayed the latency of convulsions triggered by INH	[113]
2	*Ajuga integrifolia*	L	HME	PTZ & MES	Swiss albino mice (20–30 g)	100–400	HME extract significantly delayed the latency onset of PTZ-induced convulsions at all doses (100, 200 & 400 mg/kg) and decreased the duration of tonic hind limb extension in the MES model. Unlike BU and CH fractions, the AQ fraction didn’t show any effect on latency and duration of convulsions at all doses	[149]
3	*Allium sativum*	Bu	AQ	PLC	Male adult Wistar rats (200–250 g)	100 & 300	The AQ extract demonstrated neuroprotective potential in PLC-induced neurodegeneration, mitigated the prefrontal cortex (PFC) astrogliosis. However, it didn’t decrease GLU and other neurotransmitter levels	[150]
4	*Artemisia afra*	Wh	HET	PTZ	Male BALB/c mice (22–30 g)	250–1000	Delay the mean onset of convulsion and decrease the mean duration of convulsions	[151]
5	*Asparagus africanus*	R	AQ	PLC	*Mus musculus* Swiss mice (20–29 g)	63.5–254	Decreased the duration and number of clonic and tonic convulsions. Increased the latency time of onset of clonic and tonic convulsions	[127]
6	*Azadirachta indica*	–	–	PTZ	Sprague Dawley strain male rats	100	Decrease in seizures severity by decreasing the mean onset time of jerks and protecting the brain against anoxic damage and oxidative stress (OS) due to prolonged seizures	[152]
		R	HET	PTZ & MES	Albino rats of either sex (200–250 g) & albino mice of either sex (30–50 g)	200–800	There was no significant increase in the mean duration of hind limb extension in the test groups at all doses (200, 400 & 800 mg/kg). The HET root extract was devoid of any anticonvulsant activity in rodents	[153]
7	*Balanites aegyptica*	SB	CH & HME	PTZ, MES & PLC	Male Albino Swiss mice (28–38 g) & male Albino Swiss rats (200–225 g)	200 & 400	Both solvent extracts significantly suppressed hind limb extension and delayed latency of myoclonic spasm and clonic convulsions of mice at all doses. Similarly, the CH (100 mg) and HME (100 & 200 mg) extracts delayed the latency to rearing with forelimb clonus in rats	[154]
8	*Buddleja polystachya*	L	HME	PTZ & MES	S iss albino mice (27–33 gm)	100–400	The HME extract elicited a significant anticonvulsant effect in MES (all doses) and PTZ models (200 & 400 mg/kg). The BU fractions showed a significant anticonvulsant effect in both models. In addition, the CH fractions were active against seizure-induced by PTZ (200 & 400 mg/kg). While the AQ fractions were devoid of any anticonvulsant activities in both models	[155]
9	*Carissa edulis*	RB	AQ	PTZ, PIC, STR, NMDA, INH & AMP	Swiss Albino mice (18–30 g) & Wistar albino male rats (130–220 g)	150–600	The AQ fractions protected PTZ, STR, and NMDA-induced seizures significantly at higher doses. But the AQ fractions and sub-fractions showed no effect on MES-induced seizures	[156]
			HET	PTZ & MES	Swiss Albino mice of either sex (15–24 g) & White ranger cockerels of either sex (30–41 g)	5–20	Delayed the mean onset of convulsions in mice and chicks. It exhibited a dose-dependent inhibition of the convulsion induced by MES (90% protection at 20 mg/kg)	[104]
10	*Clerodendrum myricoides*	L	HET	PTZ	Male BALB/c mice (22–30 g)	300–1200	Unlike the solvent fractions, the crude extract demonstrated a significant delay in the mean latency to onset of seizures and decrease the duration of convulsions in a dose-dependent manner	[157]
11	*Clutia abyssinica*	L	HME	PTZ & MES	Male BALB/c mice (20–30 g)	400 & 800	Though the crude extract exhibited insignificant dose-dependent delay on the onset of a seizure, it improved the survival of mice	[158]
12	*Croton macrostachyus*	SB	AQ	PIC, STR, PTZ, INH & MES	Adult male *Mus musculus* Swiss mice (19–25 g)	13–135	The crude extract prevented the mice from PIC, STR, PTZ, and MES-induced seizures. It also delayed the onset of INH-induced seizures	[140]
13	*Indigofera arrecta*	L	ME	PTZ	Zebrafish with an AB or EK strain	30–300*	The main constituent, idirubin, revealed reduction of epileptiform discharges in PTZ-treated zebrafish larvae	[144]
14	*Jatropha curcas*	L	AQ	PTZ & MES	Male albino mice (25–30 g)	100–400	Protected the mice against the MES-induced convulsion. While at 400 mg/kg, it significantly protected the mice against PTZ-induced seizures	[134]
15	*Maytenus heterophylla*	L, R & SB	ME	PIC	White Swiss albino mice (20–24 g)	50–200	The stembark extract significantly suppressed convulsions induced by PIC better than the leaf and root extracts. It also offered up to 62.5% protection against seizure at 200 mg/kg which was significant (p < 0.05) as compared to diazepam	[159]
16	*Nicotiana tabacum*	Ar	AQ & HME	PTZ	Random breed albino male mice (18–24 g)	100	Both extracts decreased the onset and severity of seizures (but it is statistically insignificant as compared to the negative control group). Both extracts decreased the mortality of PTZ-treated mice	[160]
17	*Olea europaea*	–	–	PTZ	Mice weighing (25–30 g)	20	The active constituent of *Olea europaea* leaf, oleuropein (20 mg/kg), caused a significant increase in seizure latency and a significant decrease in the whole body seizure	[161]
18	*Opuntia ficus-indica*	Fl	HME	PTZ, MES & STR	Swiss albino mice (20–25 g)	250 & 500	Protect the mice against PTZ, MES, and STR-induced seizures	[135]
19	*Pentas schimperiana*	RB	HME	PTZ & MES	Swiss albino mice (20–30 g)	100–400	The BU and ME fractions significantly inhibited the PTZ and MES-induced seizure at 400 mg/kg	[162]
20	*Pterolobium stellatum*	L	AQ & HME	PTZ & MES	Swiss albino mice (25–32 g)	100–400	The HME extract exhibited a dose-dependent increase on the latency onset of seizure against PTZ. In addition, both HME and AQ fractions demonstrated a dose-dependent reduction in duration of hind limb tonic extensions in the MES model and myoclonic seizure in the PTZ model at 400 mg/kg	[163]
21	*Ruta chalepensis*	Ar	ET	PTZ	Male Swiss albino mice (25–30 g)	10–1000	Delayed the onset of seizures and a dose-dependent suppression in the tonic phase and mortality induced by PTZ was noticed	[164]
22	*Securidaca longepedunculata*	R	AQ	STR & PIC	Albino mice of either sex (20–25 g)	100–400	The extract elicited dose-dependent increase in onset of convulsion and prolongation of the cumulative time spent in the open arms of the elevated plus maze and Y maze compared with the control	[165]
		SB	AQ	PTZ, MES & AMP	Swiss albino mice of either sex (18–25 g)	50–200	The extract afforded significant protection against the mice treated with PTZ (50 & 100 mg/kg) and MES (50 mg/kg). It didn’t attenuate AMP induced seizure though it prolonged the onset of convulasions at 100 and 200 mg/kg	[166]
23	*Sida rhombifolia*	Wh	ME	PTZ & MES	Swiss albino mice of either sex (25–30 g)	100–400	The ME crude extract significantly reduced the duration of seizures at all doses	[136]
24	*Withania somnifera*	S & R	ET	PTZ & MES	Albino Wistar rats of either sex (150–200 g)	100–300	The extracts significantly suppressed hind limb tonic extension and postictal depression in MES test groups at 300 mg/kg. Moreover, a significant reduction in the mean duration of hind limb tonic flexion, hind limb tonic extension, clonus, and stupor in PTZ test groups	[110]
25	*Xanthium stramonium*	Wh	PE	PTZ & MES	Albino Wister albino rats (150–200 g)	250 & 500	The crude extract reduced the duration of convulsions. It also delayed the onset of myoclonic spasm and clonic convulsion in albino Wister rats	[167]
26	*Zingiber officinale*	Rh	HET	PTZ	Wild type adult zebrafish of the AB strain	60^b^	The active constituent of the extract, 6-gingerol (6-GIN), effectively inhibited PTZ-induced seizures	[168]
					Adult male Swiss mice	25–200	It significantly increased the onset time of myoclonic seizures at a dose of 25–100 mg/kg and significantly prevented generalized clonic seizures	[169]

*PU* plant parts used, *L* leaf, *S* stem, *SB* stembark, *R* root, *RB* rootbark, *Wh* whole plant, *Ar* Aerial part, *Bu* bulbs, *Fl* flower and *Rh* rhizome Seizure-inducing agents *PIC* picrotoxin, *STR* strychnine, *PTZ* pentylenetetrazol, *INH* isonicotinic hydrazide acid and *MES* maximal electroshock**,** *PLC* pilocarpine, *AMP* 4-aminophylline, and *NMDA N*-Methyl-D-aspartate. Extraction solvents *AQ* aqueous, *CH* chloroform, *BU* butanol, *ET* ethanolic, *HET* hydroalcoholic/hydroethanolic, *ME* methanolic, *HME* hydromethanolic, and *PE* petroleum ether

^a^and ^b^represented the plant extract doses given in µM and µg/mL, respectively

#### In vivo pharmacological activities of crude extracts and solvent fractions

##### Single stimuli-induced seizure model

PTZ is routinely used as a stimulus to induce convulsions in different animal models by inhibiting the GABAergic neurotransmission [170]. PTZ-induced seizures are characterized by an initial ‘absence-like’ immobility, followed by brief myoclonic jerks, sustained myoclonus, and finally GTCS with a loss of the righting reflex. The subcutaneous administration of PTZ is often used to induce a seizure in mice [171] that can be employed to assess the anticonvulsant activity of MPs. The whole plant and leaf extract of *Artemisia afra* are traditionally used for the treatment of epilepsy in Ethiopia and South Africa, respectively (Table 3) [105]. Kediso et al. [151] investigated the anticonvulsant effect of the HET and solvent fractions of *Artemisia afra* whole part against PTZ-induced seizure in mice. Unlike the solvent fractions, the HET crude extract triggered a significant delay in the mean onset of convulsions (504.833 ± 62.835 s, 551.833 ± 74.69 s, and 808.333 ± 64.8 s) and a decrease in the mean duration of convulsions (17.000 ± 1.88 s, 13.000 ± 1.8 s and 7.833 ± 1.07 s) at the respective doses of 250, 500 and 1000 mg/kg. The observed activity of the crude extract might be attributed to the presence of multiple secondary metabolites in the herb. *Clerodendrum myricoides* is another MP whose leaf extract is traditionally used as an anticonvulsant in Ethiopia, Kenya, and South Africa [100, 119]. Owing this, the anticonvulsant activity of the HET and solvent fractions of the leaf extract was assessed via mice experiencing PTZ-induced seizures [157]. The HET crude extract of *Clerodendrum myricoides* at 300, 600 and 1200 mg/kg significantly delayed the mean latency in the onset of seizures (299.33 ± 30.129 s, 387.167 ± 27.6 s and 417.833 ± 31.9 s, respectively) and decrease in the duration of convulsions (27.333 ± 1.585 s, 16.833 ± 1.537 s and 10.50 ± 0.671 s, respectively) in a dose dependent manner as compared to the control group. On the other hand, the solvent fractions of *Clerodendrum myricoides* didn’t show significant anticonvulsant effect in the model.

*Ruta chalepnesis* is known for its antiepileptic activities in the traditional folklore of Ethiopia, Morocco, and Mexico [125, 126]. The ET extracts of the aerial parts of *Ruta chalepnesis* were assessed by using PTZ-induced seizure and a dose-dependent suppression in the tonic phase was observed, moreover, it reduced the mortality triggered by PTZ in the experimental animals. *Azadirachta indica* is employed in the traditional healthcare system of Ethiopia and India to treat epilepsy [128]. Kumar et al. [152] compared the antiseizure activities of Valproic acid (VPA) and *Azadirachta indica* on PTZ-induced kindling in Sprague Dawley strain male rats at 200 mg/kg and 100 mg/kg, respectively. A decrease in the mean onset time of jerks, clonus, and extensor phases was observed in VPA and *Azadirachta indica* treated groups. Moreover, an increase in glutathione reductase activity and a decrease in the activity of lipid peroxidation enzymes, glutathione S-transferase activity, catalase, and nitric oxide was observed in the same group, asserting the protective effects of VPA and *Azadirachta indica* against anoxic damage and OS of the brain due to prolonged seizures. Overall, *Azadirachta indica* demonstrated better preventive effects than VPA on PTZ-induced chemical kindling in rats. *Asparagus africanus* is a widely used plant in TM as an anti-inflammatory, antioxidant, for the treatment of CNS disorders including epilepsy. The anticonvulsant activity of the root decoction of *Asparagus africanus* was evaluated in PLC-induced SE in *Mus musculus* Swiss mice. It increased the onset time of tonic–clonic convulsions and decreased the duration and number of tonic–clonic convulsions at doses of 63.5, 127, and 254 mg/kg. The anticonvulsant activity of *Asparagus africanus* emanated from modulation of GABA (increase), GABA-T, TNF-α (decrease) levels, and inhibition of OS in the brain [127].

##### Dual stimuli-induced seizure models

MES is the second most commonly used seizure-inducing stimuli in different animal models of epilepsy next to PTZ. It is convenient to assess GTCS that can be reproduced with reliable endpoints [172]. The use of two common stimuli, PTZ and MES, in different animal models will help to better understand the pharmacological effects and the MOA of anticonvulsant agents. *Carissa edulis* is commonly used for the treatment of epilepsy in Africa especially in Ethiopia, Nigeria, South Africa, Uganda, Malawi, and Kenya [104–108]. Owing to this, the anticonvulsant activity of the rootbark of *Carissa edulis* was investigated using PTZ-induced seizure in mice and the MES test in chicks. It exhibited a suboptimal level of inhibition against seizure as compared to benzodiazepine (BZP) (100%) in the mice model. Moreover, the crude extract elicited 90% protection as compared to phenytoin (100%) at 20 mg/kg in convulsions induced by MES in chicks signifying the beneficial effect of *Carissa edulis* for the management of epilepsy and related symptoms [104]. *Clutia abyssinica* is claimed to have antiepileptic activity in traditional herbal medicine folklore of Ethiopia and Rwanda [129]. Although the HET leaf crude extract of *Clutia abyssinica* improved the mean survival time of epileptic mice, the recorded mean time of hind limb extension was not significant at 400 and 800 mg/kg as compared to the negative control group [158]. Leaves of *Jatropha curcas* have been used by TH of Ethiopia and Nigeria for the management of epilepsy. Bolanle et al. [134] examined the anticonvulsant activity of AQ leaf extract of *Jatropha curcas* in PTZ- and MES-induced seizure models. The crude extract delayed the onset of tonic leg extension and the seizure-induced mortality was inhibited in mice. Moreover, it significantly (*p* < 0.05) protected mice from MES-induced seizure at 100, 200 and 400 mg/kg. at a higher dose, 400 mg/kg, it also significantly inhibited PTZ-induced convulsions.

*Pentas schimperiana* is a MP used in Ethiopian TM for the treatment of epilepsy. Fisseha et al., [162] assessed the HME rootbark crude extract and CH, BU, and AQ fractions of *Pentas schimperiana* using PTZ and MES-induced seizure models at doses of 200 and 400 mg/kg. As compared to the control group, the ME and BU fractions, at 400 mg/kg, demonstrated significant (*p* < 0.001) anticonvulsant activities in both models. In addition, the CH fraction exerted significant (*p* < 0.001) seizure control in PTZ treated mice whereas the aqueous fraction was devoid of significant antiepileptic activities in both models. In general, the alkaloids, flavonoids, saponins, tannins, phenols, steroids, and terpenoids present in the rootbark may be ascribed to the observed seizure control in mice. *Sida rhombifolia* is a plant commonly prescribed for the treatment of epilepsy by the THs of Ethiopia and India [136]. The ME crude extract of the whole part of *Sida rhombifolia* was examined PTZ and MES-induced seizure in mice at 100, 200, and 400 mg/kg. The result reiterated that the ME crude extract of 100, 200, and 400 mg/kg significantly suppressed the duration of seizure as compared to the control group in both models. *Xanthium stramonium* is a famous MP in China due to its widespread healthcare prominence. It is also used for the treatment of epilepsy in Ethiopia and India [141]. Owing to this, Kumar et al. [167] screened the anticonvulsant activity of the PE whole plant extract of *Xanthium stramonium* against PTZ and MES-induced seizure models in albino Wistar rats at a dose of 250 and 500 mg/kg. It increased the latency onset of myoclonic spasms and clonic convulsions in PTZ-treated groups. In addition, it also reduced the mean duration of the exterior phase significantly as compared to the control group in the MES test. The root of *Azadirachta indica* was used in herbal formulations prepared to treat epilepsy in different countries. The in vivo anticonvulsant assessement done on PTZ-induced seizure in mice and MES-induced seizure in Albio rats indicated that the ET root extract has no significant effect on the mean duration of limb extension, mean onset of convulsions and mean number of convulsions at a dose of 800 mg/kg as compared to the control group [153].

##### Multiple stimuli-induced seizure models

Multiple stimuli-induced seizure models provide better information about the effect of drugs or a plant extract in the target experimental animals. The depth and breadth of data obtained in such multiple seizure models can shed light on the different aspects of the plant extract under consideration: MOA, potential targets for antiepileptic interventions, possible bioactive compounds, etc. In addition to PTZ and MES, one or more of the following stimuli such as INH, PIC, PLC, NMDA, STR, AMP, and BIC are used to induce convulsions (in experimental animals) in epilepsy research. Traditional herbalists of Ethiopia, Tanzania, and Kenya [113, 114] have faith in the curative effect of *Acalypha fruticosa* for the treatment of epilepsy. Govindu et al., [113] assessed the anticonvulsant activity of the CH crude extracts of the aerial parts of *Acalypha fruticosa* using PTZ, MES, and INH-induced seizures in Swiss albino mice at doses of 30, 100, and 300 mg/kg. The result confirmed the potential of the crude extract to suppress seizures triggered by MES in a dose-dependent pattern. At 300 mg/kg, as compared to diazepam (4 mg/kg) the extract demonstrated more pronounced anticonvulsant activity. It also inhibited the PTZ-induced seizures better than the positive control, phenobarbitone sodium. While in the INH model, it delayed the onset of convulsions in a dose-dependent manner but failed to protect the mice from seizure-induced mortality. *Balanites aegyptiaca* is used traditionally in Ethiopia, Mali, Saudi Arabia [116, 117], and India to treat epilepsy. Hence, HMET and CHL extract of stembark of *Balanites aegyptiaca* were assessed using PTZ, MES-induced convulsions, and PLC-induced SE in rats [154]. Both the HME and CH extract at 200 and 400 mg/kg significantly delayed the onset of myoclonic spasm and clonic convulsions as well as significantly reduced the duration of hind limb extension in PTZ and MES models. In the PLC model, the CH extract (100 mg) and HME extract (100 and 200 mg) delayed the latency to rearing with forelimb clonus significantly.

*Carissa edulis* is popular in African countries such as Ethiopia, Nigeria, South Africa, Uganda, Malawi, and Kenya [104–108] for its beneficial effect in the management of epilepsy by herbalists or TH. The anticonvulsant activity of the AQ fractions (150, 300, and 600 mg/kg) and sub-fractions (250, 500, 500, and 1000 mg/kg) of the rootbark extract was examined using PTZ, PIC, NMDA, INH, STR, and AMP-induced seizures in mice. The AQ fraction and sub-fractions suppressed 50% and 16.67% of PTZ-induced convulsions. Similarly, the AQ fraction experienced 33.33% and 16.67% protection against strychnine and NMDA seizure models, respectively. Moreover, the AQ fractions elicited 66.67–33.33% protection against AMP-induced seizures at doses of 150 and 600 mg/kg. However, the AQ fractions and sub-fractions did not affect MES-induced seizures. *Croton macrostachyus* is a common tree used to treat epilepsy in Ethiopia and Cameron [140]. Bum et al. [140] employed MES, STR, PTZ, PIC, and INH-induced seizure models to evaluate the anticonvulsant activity of AQ stembark extract of *Croton macrostachyus* in *Mus musculus* Swiss mice. The extract protected 60, 80, 80, and 80% of mice from MES, PTZ, PIC, and STR-induced convulsions, respectively even at an initial dose of 34 mg/kg. It also increased the latency onset of seizures in INH-treated mice. Overall, the result suggested that *Croton macrostachyus* may have a promising effect in secondary GTCS and primary generalized seizures in humans. *Opuntia ficus-indica* commonly known as cactus pear is used in the treatment of epilepsy in Ethiopia and India [135]. The in vivo anticonvulsant activity of the flower ME extract was assessed using Swiss Albino mice. The ME extract produced significant inhibition against PTZ, MES, and STR-induced convulsion at 250 and 500 mg/kg. There was an increase in noradrenaline and dopamine level in the mice's brains due to the avoidance of MES-induced convulsions.

### In vivo pharmacological activities of isolated compounds/constituents

*Indigofera arrecta* is a common MP used by the indigenous inhabitants of Ethiopia, Nigeria, Congo, and South Africa [100, 122]. Bioassay-guided fractionation of *Indigofera arrecta* in zebrafish model results in the identification of indirubin and 6-bromoindirubin-3ꞌ-oxime (BIO-acetoxime), compounds with glycogen synthase kinase (GSK)-3 inhibition activity demonstrated significant anticonvulsant activity in PTZ-induced seizure in zebrafish larvae. Moreover, they also showed significant antiseizure activity in the PLC rat model limbic seizure and the 6-Hz refractory seizure mouse model, demonstrating GSK-3 inhibition as a potential therapeutic target for epilepsy. *Olea europaea* is among the known MPs used for the management of epilepsy in Ethiopia and Kenya [108]. Oleuropin, a secondary metabolite extracted from the leaves of *Olea europaea*, elicited a significant increase in seizure latency and a significant decrease in total frequencies of head ticks, head and upper limbs seizures, frequent spinning and jumping, and tonic seizures in PTZ kindling of seizure in mice. Oleuropin treated groups (20 mg/kg) showed downregulation of genes responsible for the expression of IL-1 without change in GLT-1 levels. The significant antepileptic activity of oleuropin may be attributed to its antioxidant and antiinflammatory activities making it an ideal pharmacophore for the synthesis of AEDs. *Zingiber officinale* is another most frequently used medicinal herb in different parts of the world. For instance, in Ethiopia and Japan *Zingiber officinale* is used for the management of epilepsy [137]. Its HET extract of rhizome has demonstrated anticonvulsant activity in rodent seizure models [169, 173]. Gawel et al., [168] also proved the anticonvulsant effect of ME crude extract using a PTZ-induced seizure in zebrafish larvae. Inspired by its activity, the group also isolated the major constituent of *Zingiber officinale* rhizome, 6-gingerol (6-GIN) that exerted dose-dependent antiseizure activity in PTZ-induced hyperlocomotion assay in zebrafish larvae. Rigorous experimental procedures and molecular docking analysis in human NR2B-containing NMDA receptors suggested that the antiepileptic activity of 6-GIN may be partly mediated by restoring the balance between GABA and GLU in the epileptic brains. In general, the in vivo anticonvulsant activity of the aforementioned MPs resonated the potentials of herbal formulations in the healthcare system of different countries. Although most of the antiepileptic MPs claimed by THs were not screened for their anticonvulsant effects through suitable seizure models, this review partly documented the strong association that exist between the indeginous knowledge of THs and pharmacological activities of MPs used to treat epilepsy and related symptoms in Ethiopia and other parts of the world.

### Toxicity profiles of antiepileptic or anticonvulsant medicinal plants

#### Acute toxicity profiles of medicinal plants

Acute toxicity study of plant extracts is performed to the assess the potential inherent toxicity that may be displayed in a short period of time upon a single dose exposure mostly via the oral route as it is considered as a viable route for accidental human exposure for hazardous substances and it allows for hazard classification of test substances [174]. The leaf part of *Artemisia afra*, *Azadirachta indica*, *Brucea antidysenterica*, *Buddleja polystachya*, *Eucalyptus Globulus*, *Gloriosa superba*, *Maytenus heterophylla*, *Nicotiana tabacum*, and *Ocimum lamiifolium* are commonly used for the preparation of remedies used to treat epilepsy and related symptoms in Ethiopia. The acute toxicity studies conducted in the crude extracts, essential oils and bio-oils recapped the absence of gross behavioral, physical changes and signs of overt toxicity such as lacrimation, urination, muscle weakness and convulsions in different animal models [175–181]. As depicted in Table 5, relatively higher LD_50_ value greater than 5000 mg/kg of body weight were recorded for *Artemisia afra*, *Azadirachta indica*, *Gloriosa superba*, and *Nicotiana tabacum* extracts. In addition, the EO of *Eucalyptus Globulus*, and HET extract of *Maytenus heterophylla* 2.5 mL/kg and > 1200 mg/kg, respectively demonstrating the safety profiles of single dose of the plant extracts. Furthermore, the roots of *Asparagus africanus*, *Biophytum umbraculum*, *Capparis tomentosa*, and *Withania somnifera* are believed to be rich in bioactive chemicals characterized by attenuating convulsions. Their crude extracts and solvent fractions were devoid of any inherent acute toxicity symptoms at a single dose greater than 2000 mg/kg body weight [182–185]. The AQ and HME stembark extract of *Croton macrostachyus* (LD_50_ > 5000) and the ET rootbark crude extract of *Carissa edulis*, (LD_50_ ⁓3,808) were found to be safe [186, 187], consequently, the experimental animals manifested neither visible signs of lacrimation, loss of appetite, tremors, hair erection, salivation, diarrhea and convulsion nor mortality in the study period at the estimated doses equivalent to LD_50_ values. According to Globally Harmonized Classification System (GHCS) for chemical substances and mixtures, synthetic chemicals and plant extracts having an LD_50_ > 2000 mg/kg of body weight is considered as safe [188]. This reiterated the relative safety profiles of most MPs used to treat epilepsy and related symptoms in Ethiopia.

**Table 5:** Acute toxicity profiles of some MPs employed in the treatment of epilepsy and related symptoms

No.	Scientific name	PU	Extract	Animal models	Acute toxicity studies			Refs.
					Doses (mg/kg)	LD_50_ (mg/kg)	Treatment outcomes	
1	*Ajuga integrifolia*	R	HME	Swiss albino male mice (20–30 g)	2000	> 2000	Neither mortality of mice nor any signs of toxicity (behavioral, neurological, autonomic, or physical changes) was observed at 2000 mg/kg of body weight	[189]
2	*Allium sativum*	Bu	AQ	Wistar rats (⁓115–126 g)	100, 1000, 2500 & 5000	> 5000	No death was recorded at all doses. The rats treated with 5000 mg/kg of body weight experienced cardiac problem and disorientation	[190]
3	*Artemisia abyssinica*	Ar	ET	Swiss albino mice (25–30 g)	500, 1000 & 3000	> 3000	The mice did not show visible toxicity, although at 3000 mg/kg a decreased in locomotor activity was observed	[191]
4	*Artemisia afra*	L	AQ	Female adult Swiss albino mice (25–30 g)	200, 700, 1200, 2200, 3200, 4200 &5000	> 5000	Mild toxicities like anxiety and piloerection were observed at higher doses (≥ 3200 mg/kg) that disappear in the wash out periods. No mortality in mice was recorded at all doses	[175]
		L	ET, DCM & HX	Swiss albino mice (20–22 g)	1000, 2000 and 2500	> 2500	Loss of appetite, hypoactivity, lethargic, dizziness that disappeared in the washout period was noticed in mouse treated with DCM extract at 2500 mg/kg	[192]
5	*Asparagus africanus*	R	HET & BU	Swiss albino mice (20–25 g)	1000, 3000 & 5000	> 5000	There was no dose-dependent behavioral change, weight change and mortality in mice treated single dose BUT fraction orally	[182]
6	*Azadirachta indica*	L	AQ	Female BALB/c mice (average mass of 30 g)	1250, 2500 & 5000	> 5000	The mice treated with the extract were devoid of weight/hair loss, allergy, or other symptoms of discomfort	[176]
7	*Balanites aegyptiaca*	SB	AQ	Fishes	17.5, 20, 22.5 & 25^a^	⁓18.99–20.72^a^	*B. nurse*, *L. intermedius* and *L. bynni* fish species treated with the extract suffered from the debilitating toxic effect	[193]
8	*Biophytum umbraculum*	R	AQ, BU & CH	Female Swiss Albion mice (22–30 g)	2000	> 2000	There was no behavioral change, weight change and mortality in mice treated single dose of all fractions	[183]
9	*Brucea antidysenterica*	L	AQ, ME & CH	Swiss albino mice (27–36 g)	500, 1000 & 2000	–	The extracts lack visible signs of acute toxicity and mice fatality till the dose of 1000 mg/kg. But, at the dose of 2000 mg/kg it caused mortality in all mice with in 24 h	[194]
10	*Buddleja polystachya*	L	HME	Female Sprague–Dawley rats (150–200 g)	2000	–	There was no visible sign of skin reaction, inflammation, erythema, irritation or redness, and any adverse reaction in rats	[177]
11	*Calpurnia aurea*	L	AQ & HME	Female Swiss albino mice	5000	> 5000	The mice were devoid of gross behavioral or physical changes and signs of overt toxicity such as lacrimation, urination, muscle weakness and convulsions	[186]
12	*Capparis tomentosa*	R	HME	Male Swiss Albino mice (25–38 g)	2000, 3000 & 5000	> 2000	The mice showed signs of slight rigidity and sleepy activity at higher doses of extract (3000 and 5000 mg/kg). No mortality was recorded at all doses	[184]
13	*Carissa edulis*	L	AQ	Wistar albino rats of either sex	2000	> 2000	The rats showed no gross behavioral or physical changes and signs of overt toxicity	[195]
		RB	ET	Wistar albino rats (124–220 g) & Swiss mice (16–35 g)	10, 100 & 1000	⁓3808	None of the mice and rats orally treated with the extract manifested signs of toxicity except death at the dose of 5000 mg/kg (in both species)	[187]
14	*Caylusea abyssinica*	L	HME	Male Swiss albino mice (20–30 g)	2000	> 2000	The mice didn’t experience any behavioral, neurological, autonomic or physical changes	[196]
15	*Clerodendrum myricoides*	R	AQ	Swiss albino mice of either sex (25–30 g)	1134	–	Behavioral changes such as horripilation, difficulty in breathing, grooming, and asthenia followed by death was noticed in mice treated with 1134 mg/kg	[197]
16	*Croton macrostachyus*	R	HME	Female Swiss Albino mice (25–28 g)	2000 & 5000	> 5000	The mice showed no visible signs of lacrimation, loss of appetite, tremors, hair erection, salivation, diarrhea and convulsion	[198]
		SB	AQ & HME	Female Swiss albino mice	5000	> 5000	The mice were devoid of gross behavioral or physical changes and signs of overt toxicity such as lacrimation, urination, muscle weakness and convulsions	[186]
		SB	HME, AQ & ETAc	Female Swiss albino mice	2000	> 2000	None of the mice treated with crude extract or solvent fractions showed problems in breathing, alertness, motor activity, restlessness, diarrhea and convulsions	[199]
17	*Cucumis ficifolius*	R	HME & CH	Swiss albino mice (25–30 g)	125, 250, 500 & 2000	> 2000	There were no mortality and signs of overt toxicities at a dose of 2000 mg/kg of body weight	[200]
18	*Echinops kebericho*	Tu	EO	Swiss albino mice (18–26 g)	300 & 2000	> 2000	Though the mice showed piloerection, muscle spasm and apathy immediately after administration, there were no significant treatment-related morbidities	[201]
		Tu	AQ	Wistar albino rats (250–350 g)	300, 2000 & 5000	> 5000	The rats experienced piloerection, muscle twinge, and lethargy after the treatment with the extract (5000 mg/kg) which disappeared after 5 h. But, there were no treatment related morbidity and mortality at 5000 mg/kg	[202]
19	*Embelia schimperi*	Fr	HET	Female Wistar rats (180–210 g)	400, 1000, 2000, 3000, 4000 & 5000	> 5000	The extract didn’t elicit prominent signs of toxicity and any mortality in rats in the study period	[203]
20	*Eucalyptus Globulus*	L	EO	Swiss albino mice of either sex (23–30 g)	2, 2.5, 3 & 3.5^b^	2.5^b^	The mice treated with the essential oil showed restlessness, debilitation, reduced food and water intake and piloerection which disappeared in the washout period after treatment with ≥ 2.5 mL/kg	[178]
21	*Fagaropsis angolensis*	SB	HME, AQ, BU & CH	Adult male Swiss albino mice (25–30 g)	2000	≥ 2000	Neither mortality nor any signs of toxicity were observed in mice treated with both extracts at 2000 mg/kg body weight	[204]
22	*Foeniculum vulgare*	Fr	ET	Swiss labial mice (25–28 g)	500, 1000 & 3000	≥ 3000	The extract didn’t trigger mortality of mice and overt toxicity except reduced locotmotor activity and piloerection at 3000 mg/kg of body weight	[205]
23	*Gloriosa superba*	L	AQ	White male Wistar rats (200–250 g)	⁓121, 364, 1091 & 3274	> 1500	The rats experienced treated with colchicine of standardized *Gloriosa superba* extract showed no visible sign of overt toxicity	[206]
		L	HME	Non-pregnant Wistar rats (120–140 g)	200 & 5000	> 5000	There were no visible overt signs of toxicity at 5000 mg/kg. No morbidity or mortality was observed in the rats treated groups at both doses	[179]
24	*Justicia schimperiana*	L	HME	Swiss albino mice (18–30 g)	2000	> 2000	The extract didn’t trigger signs of overt toxicity. Moreover, no mortality of mice was recorded in the study period	[207]
		L	HME	Female adult Wistar rats (180–200 g)	2000	> 2000	Rats showed no formation of edema or erythema. No signs of toxicity as well as no mortality were noted during the study period	[208]
25	*Maytenus heterophylla*	L	HET	Male CD-6 mice (35–40 g)	1200	> 1200	The mice treated with the extract were devoid of physical and behavioral changes at 1200 mg/kg	[180]
26	*Maytenus senegalensis*	RB	ET	Swiss albino mice (18–22 g)	200, 300, 400, 800 & 1600	> 1600	The mice treated with the extract were devoid of physical and behavioral changes at 1600 mg/kg	[209]
		SB	HET	Theiller’s albino mice of either sex	1000, 2000, 3000, 4000 & 5000	> 5000	The mice treated with the extract were devoid of physical and behavioral changes at 5000 mg/kg	[210]
		L & S	HET	Male CD-6 mice (35–40 g)	1200	–	The mice treated with leaf extract exhibited some signs of overt toxicity. In addition, the stem extract caused pronounced toxicity at 1200 mg/kg	[180]
27	*Myrica salicifolia*	R	HME	Non-pregnant female mice	2000	> 2000	There are no visible signs of overt toxicity and mortality in mice treated with the extract at 2000 mg/kg	[211]
28	*Nicotiana tabacum*	L	Bio-oil	Female Wistar rats (130–140 g)	5000	> 5000	The rats exhibited no significant change in the body weight and behavior. In addition, there was no mortality of rats in the study period	[181]
29	*Ocimum lamiifolium*	L	ME	Swiss albino mice (27–36 g)	500, 1000 & 2000	≥ 2000	The crude extract didn’t trigger gross visible signs of acute toxicity such as urination, hair erection, lacrimation, and reduction in feeding activity	[194]
30	*Olea europaea*	L	ET	Wistar rats of either sex (150–200 g)	2000	≥ 2000	Oral administration of the extract didn’t cause any mortality or sign of toxicity at 2000 mg/kg of body weight during the study period	[212]
31	*Opuntia ficus-indica*	S	HET	White Sprague Dawley rats either sex	500, 1000 & 2000	–	The rats exhibited no genotoxicity at all treatments regimens even at the maximum dose of 2000 mg/kg	[213]
		Se	HX (fixed oil)	Mus musculus mice (20–30 g)	10, 20, 30, 40, 50, 60 & 70^b^	43^b^	The mice suffered from immediate agitation and behavioral perturbations with temporary writhing, followed by a quiet attitude period and sedation	[214]
32	*Pentas schimperiana*	L	AQ & HME	Swiss Albino mice of either sex (20–33 g)	1000, 2000 & 5000	> 4000	The mice experienced no visible change in behavior such as restlessness, motor activity, breathing and diarrhea. Moreover, there was no mortality recorded at 5000 mg/kg	[215]
33	*Podocarpus falcactus*	Ap	AQ	Female Sprague Dawley rats (260–300 g)	2000	> 2000	The rats showed neither mortality nor gross behavioral changes and mortality at 2000 mg/kg of body weight	[216]
34	*Ruta chalepensis*	Ar	ET	Male Swiss albino mice (25–30 g)	1600, 3000 & 5000	> 5000	The extract didn’t trigger mortality nor macroscopic tissue injury or weight loss at 5000 mg/kg per body weight	[164]
35	*Rhus vulgaris*	SB	AQ,	Female Swiss albino mice (18–26 g)	50, 300 & 2000	> 2000	The mice were devoid of changes in general appearance and behavioral patterns. In addition, there was no mortality or gross pathology in any organ at necropsy	[217]
36	*Securidaca longepedunculata*	L, S & R	AQ & ME/CH (1:1)	Swiss female mice (20–22 g)	50, 300 & 2000	> 2000	The AQ total extracts of leaves and stembark did not show any change in behavior following administration of the crude extracts at 2000 mg/kg of body weight	[218]
37	*Sida rhombifolia*	Ar	ET	Adult male Wistar albino rats (180–220 g)	2000	> 2000	There were no visible overt signs of toxicity and mortality in rats treated with 2000 mg/kg of the extract	[219]
		Ar	HME	Albino Wistar rats (102–134 g)	4000, 8000, 12000 & 1600	> 8000	The rats exhibited slight changes in general behavior such as sow response to external stimuli, stretching and sluggishness	[220]
		R	AQ	Sprague Dawley rats of either sex (130–190 g)	5000	> 5000	The rats experienced neither overt toxicity signs nor mortality at a single dose of 5000 mg/kg	[221]
38	*Syzygium* *guineense*	L	HME	Wistar rats of either sex (120–140 g)	2000 & 5000	> 5000	In the acute toxicity study, rats treated with 2000 mg/kg and 5000 mg/kg showed no toxicological signs observed on behavior, gross pathology, and body weight of rats	[179]
39	*Teclea nobilis*	Wh	HME & AQ	Male Swiss mice (⁓20 g)	1000, 2000, 3000, 4000 & 5000	> 5000	The extract was devoid of any overt toxicities at 5000 mg/kg of body weight. Moreover, there was no mortality recorded in the study period	[222]
40	*Vernonia amygdalina*	L	AQ & HME	Female Swiss albino mice	5000	> 5000	The mice exhibited no signs of overt toxicity such as lacrimation, urination, muscle weakness, sedation and convulsions at 5000 mg/kg	[186]
		L	AQ & ET	Albino Wistar rats (200–250 g)	2000	> 2000	The extracts triggered no significant effect on the biochemical and hematological parameters of treated rats (no lesions were also observed in the liver and kidneys histologically)	[223]
41	*Withania somnifera*	R	ME	Wistar rats	5000, 1000 & 2000	> 2000	The rats didn’t experience any organ atrophy, hypertrophy, and degenerative or infiltrative lesions even at 2000 mg/kg	[185]
42	*Zingiber officinale*	R	HX (fixed oil), EO	Swiss albino mice (23–26 g) and Wistar rats (150–170 g)	0.02, 0.04, 0.06, 0.08 and 0.1^b^ mL/kg for fixed oil; 0.2, 0.4, 0.6, 0.8, 1.0, 2.0, 4, 6, 8 and 10^b^ for EO	–	Observed cardinal signs of toxicity for both oils were decreased motor activity, convulsion and paralysis. In addition, mortality of experimental animals was noticed in both fixed-oil (0.2 mL/kg) and EO treated group	[224]

*PU* plant parts used, *Ap* apex, *L* leaf, *S* stem, *Se* seeds, *SB* stembark, *R* root, *RB* rootbark, *Ar* Aerial part, *Bu* bulbs, *Tu* tuber and *Rh* rhizome, Extraction solvents, *AQ* aqueous, *CH* chloroform, *BU* butanol, *DCM* dichloromethane, *ET* ethanolic, *ETAc* ethyl acetate, *HX* hexane, *HET* hydroalcoholic/hydroethanolic, *ME* methanolic, *HME* hydromethanolic, *PE* petroleum ether and *EO* essential oil

^a^and ^b^represented the plant extract doses and LD_50_ values are given in mg/L and mL/kg, respectively

#### Subacute toxicity profiles of medicinal plants

Acute toxicity studies provide preliminary data about the safety profiles of a single dose of chemical agents [225], consequently, it is considered as shallow and sometimes misleading. Better information about the safety of chemicals of synthetic and natural origin can be obtained from the subacute toxicity studies, which involve repeated administration of the chemical agent under consideration. In subacute toxicity assessments, weight loss of experimental animals is an important variable that can be attributed to harmful effects of test substances [179]. A weight loss, that may be attributed to the anti-nutritive and malabsorption effect of chemical agents, that amount to ≥ 10% can be considered a sign of toxicity even in the absence of other changes on target organs, haematological or biochemical effects [226]. The subacute toxicity of plant-based materials including crude extracts, solvent fractions, bio-oils, essential oils, etc. was evaluated through repeated administration a specific dose in different animal models with the intention of assessing its accumulation in the body with gradual effects on tissues and organs [188]. In this regard, Loha et al. [179] assessed the subacute toxicity of HME leaf extract of *Syzygium guineense* in rats at 500 and 1500 mg/kg of body weight. Herein, the rats were devoid of significant change on behavior, gross pathology, body weight, and hematological and biochemical parameters, asserting the safety profile of the leaf extract at a repeated dose of 1500 mg/kg. In addition, subacute toxicity study was conducted on EO obtained from *Echinops kebericho* tuber at the doses of 100, 200 and 400 mg/kg [201]. The EO treated groups did not experience significant dose-dependent alterations in body weight, clinical chemistry parameters and relative organ weights. Deyno et al. [202] confirmed that *Echinops kebericho* decoction was well tolerated up to the dose of 600 mg/kg body weight as food consumption, body weight, organ weight, hematology, clinical chemistry, and histopathology did not show significant alterations between control and treatment groups.

Moreover, subacute toxicity studies conducted on the different extracts of antiepileptic or anticonvulsant MPs such as *Allium sativum* (AQ bulb extract at 300 mg/kg) [190], *Artemisia abyssinica* (ET extract of the aerial part at 3000 mg/kg) [191], *Artemisia afra* (AQ leaf extract at 1800 mg/kg) [175], *Asparagus africanus* (HET and BU root extracts) [182], *Azadirachta indica* (AQ leaf extract at 1000 mg/kg) [176], *Capparis tomentosa* (HME root extract at 1000 mg/kg) [184], *Eucalyptus Globulus* (EO of leaf at 2 mL/kg) [178], *Olea europaea* (ET leaf extract at 400 mg/kg) [212], *Opuntia ficus-indica* (HET stem extract at 2000 mg/kg) [213], *Myrica salicifolia* (HME root extract at 400 mg/kg) [211], *Sida rhombifolia* (AQ root extract at 1200 mg/kg) [221], and *Withania somnifera* (ME root extract at 2000 mg/kg) [185] clearly asserted their safety profiles at the respective maximum doses per body weight as manifested by the absence of significant treatment related variations in clinical observations, ophthalmic examination, body weight gain, feed consumption, clinical pathology evaluation, organ weight, and so on. On the other hand, notable discomforts or mild signs of toxicities were observed on rats treated with some MPs utilized in the management of epilepsy and related symptoms. For instance, Zewdu et al. [203] conducted subacute toxicity study on the HET fruit extract of *Embelia Schimperi* in Wistar rats at doses of 400 and 1600 mg/kg body weight. The result revealed that chronic administration of the extract (1600 mg/kg) was not significantly associated with body weight loss and organ weights such as liver and kidney. Some haematological and biochemical parameters such as platelets and AST concentration were significantly increased which may be attributed to inflammation of liver and kidney tissue upon repeated dose exposure, stressing the mild toxicity of the fruit extract of *Embelia Schimperi* at a dose of 1600 mg/kg or higher. In addition, fixed oil of *Zingiber officinale* root was found to have inherent propensity to trigger a range of toxicities (0.4 mL/kg) including hypertrophy of the liver, kidneys, lungs and spleen, cellular toxicity and oxidative stress following 60-day subchronic toxicity study [224]. Similarly, repeated administration of *Clerodendrum myricoides* AQ root extracts in mice causes reduction in body weight gain, damage to the liver and kidney and changes in some hematological and biochemical parameters in mice. The research group also reported the significant body weight loss of the AQ leaf extract of *Croton macrostachyus* at 1000 mg/kg in the treated groups [227].

#### Developmental toxicity profiles of medicinal plants

Prenatal development is comprised of pre-embryonic, embryonic and fetal stages. The embryonic stage is a critical period where organs of the embryo as well as the placenta can be damaged if exposed to toxic agents directly or indirectly. At times, toxic agents may cross the compromised placental membrane and elicit debilitating effect on the developing embryonic/fetal tissues [228]. The developmental toxicity studies of crude extracts, solvent fractions and/or essential oils has paramount healthcare implications for PS consumed by pregnant women for therapeutic as well as nutritional purpose [229]. In this regard, the effect of some MPs that are frequently employed to relive seizure in patients with epilepsy on prenatal growth (developing embryos and fetuses) are assessed by using different animal models. For instance, the developmental effect HET fruit extract of *Embelia schimperi* on embryo and fetuses was investigated by using Wistar albino rats and the result echoed that the crude extract was devoid of a significant toxic effect on embryonic and fetal development indices (in the period of organogenesis) at a dose of 1000 mg/kg body weight [230]. Similarly, the HET leaf extract of *Syzygium guineense* was evaluated at a dose of 250, 500 & 1000 mg/kg in the same animal model and the extract didn’t compromise the number of implantations, fetal resorptions, live births, and stillbirths in the same animal model though there was dose-dependent decrease in the weight of the fetuses and the placentae [228]. Abebe et al., also assessed the teratogenic potentials of the HET leaf extract of *Gloriosa superba* on Wistar albino rats (220–240 g) at a dose of 250, 500 and 1000 mg/kg of body weight. The crude extract was devoid of any significant teratogenic effects on rat embryos/fetuses up to 500 mg/kg but influenced the growth of embryos at 1000 mg/kg of body weight as manifested by diminished crown-rump length, decreased number of somites and morphological scores [231]. Moreover, the teratogenic effect of the HME leaf extract of *Catha edulis* was investigated on pregnant Wistar albino rats at a dose of 250, 500 & 750 mg/kg of body weight. The result echoed that khat extract presented dose-dependent toxicity in rat embryo and fetuses such as cytolysis, decidual hypoplasia and atrophy [232]. Overall, the aforementioned acute, subacute and developmental toxicity results witnessed the safety of MPs utilized in the management of epilepsy and related symptoms in Ethiopia.

## Phytochemistry of medicinal plants with anticonvulsant activities

MPs have been used as a source of pharmaceutical agents for numerous indications and among small molecule drugs approved between 1981 and 2010, more than half were derived from natural products, mainly plants [233]. Cannabidiol is the first AED of plant origin (extracted from *Cannabis sativa*) approved by the United States Food and Drug Administration (FDA) in 2018 for the treatment of two rare and severe forms of epilepsy, Dravet syndrome and Lennox-Gastaut syndrome [234]. In LMICs, MPs are consistently used for the treatment of several CNS disorders including epilepsy partly due to their tolerable side effects and impressive efficacy [18]. Most of the MPs prescribed for epilepsy treatment by THs have shown promising anticonvulsant activity against stimuli-induced in vitro and in vivo seizure models [8, 146]. Generally, phytochemical constituents of MPs which belong to the class of alkaloids, flavonoids, terpenoids, glycosides, coumarins, etc. are implicated in the amelioration of convulsions as confirmed by different animal models [235]. They act on different targets such as synapses, receptors, and associated neuronal pathways, ion channels, immune system, inflammatory mediators, glial cells, etc. implicated in the occurrence and progression of epileptogenesis [235]. The antiepileptic activity of MPs discussed before was mostly based on the crude extract or EO rather than isolated active compounds. Consequently, it is difficult to gain full insight into the active constituents, possible targets, effective doses, and MOA of antiepileptic PMs. This section highlights the phytochemical constituents of MPs claimed by THs for their curative effects against epilepsy and proved by in vivo experiments using different stimuli-induced seizure models. It is noteworthy to mention that the bioactive compounds or secondary metabolites of MPs discussed below are obtained from independent phytochemical screening or investigations done elsewhere regardless of their use citations. In this regard, several phytoconstituents with profound anticonvulsant activities were found in different parts including leaf, stem, stembark, root, rootbark, rhizome, flower, aerial and whole part, etc. of the reported antiepileptic MPs (Table 6). Flavonoids and terpenoids (including monoterpenes, sesquiterpenes, diterpenes, triterpenes) are the most frequently encountered phytochemicals in the antiepileptic MPs discussed in previous sections.

**Table 6:** Phytoconstituents of MPs with in vivo antiepileptic/anticonvulsant activities

No.	Scientific name	Active compounds	Refs.
1	*Ajuga integrifolia*	Apigenin and quercetin	[236]
2	*Allium sativum*	Quercetin	[237]
3	*Artemisia afra*	Borneol, camphor, eucalyptol, eugenol, *p*-cymene, phytol, α-terpineol, and β-caryophyllene	[238, 239]
4	*Azadirachta indica*	Phytol	[240]
5	*Balanites aegyptica*	Apigenin, quercetin and rutin	[241]
6	*Buddleja polystachya*	Camphor, phytol, rutin, ursolic acid and α-terpineol	[242–244]
7	*Carissa edulis*	Lupeol and rutin	[245, 246]
8	*Croton macrostachyus*	Lupeol, linalool, *p*-cymene, α-terpineol, and β-caryophyllene	[247–249]
9	*Jatropha curcas*	Lupeol, phytol and rutin	[250–252]
10	*Maytenus heterophylla*	Lupeol	[253]
11	*Nicotiana tabacum*	Apigenin, lupeol and quercetin	[254, 255]
12	*Olea europaea*	Apigenin, oleuropein and quercetin	[256]
13	*Opuntia ficus-indica*	Quercetin and rutin	[257]
14	*Ruta chalepensis*	Borneol, camphor, carvacrol, linalool, menthol, pulegone, quercetin, rutin and α-terpineol	[258–260]
15	*Sida rhombifolia*	Lupeol	[261]
16	*Xanthium stramonium*	Borneol, lupeol, *p*-cymene, quercetin, β-caryophyllene	[262, 263]
17	*Zingiber officinale*	6-gingerol, borneol, camphor, citral, citronellol, linalool, *p*-cymene, α-terpineol and β-caryophyllene	[168, 264]

### Flavonoids with anticonvulsant activities

**Fig. 2:**
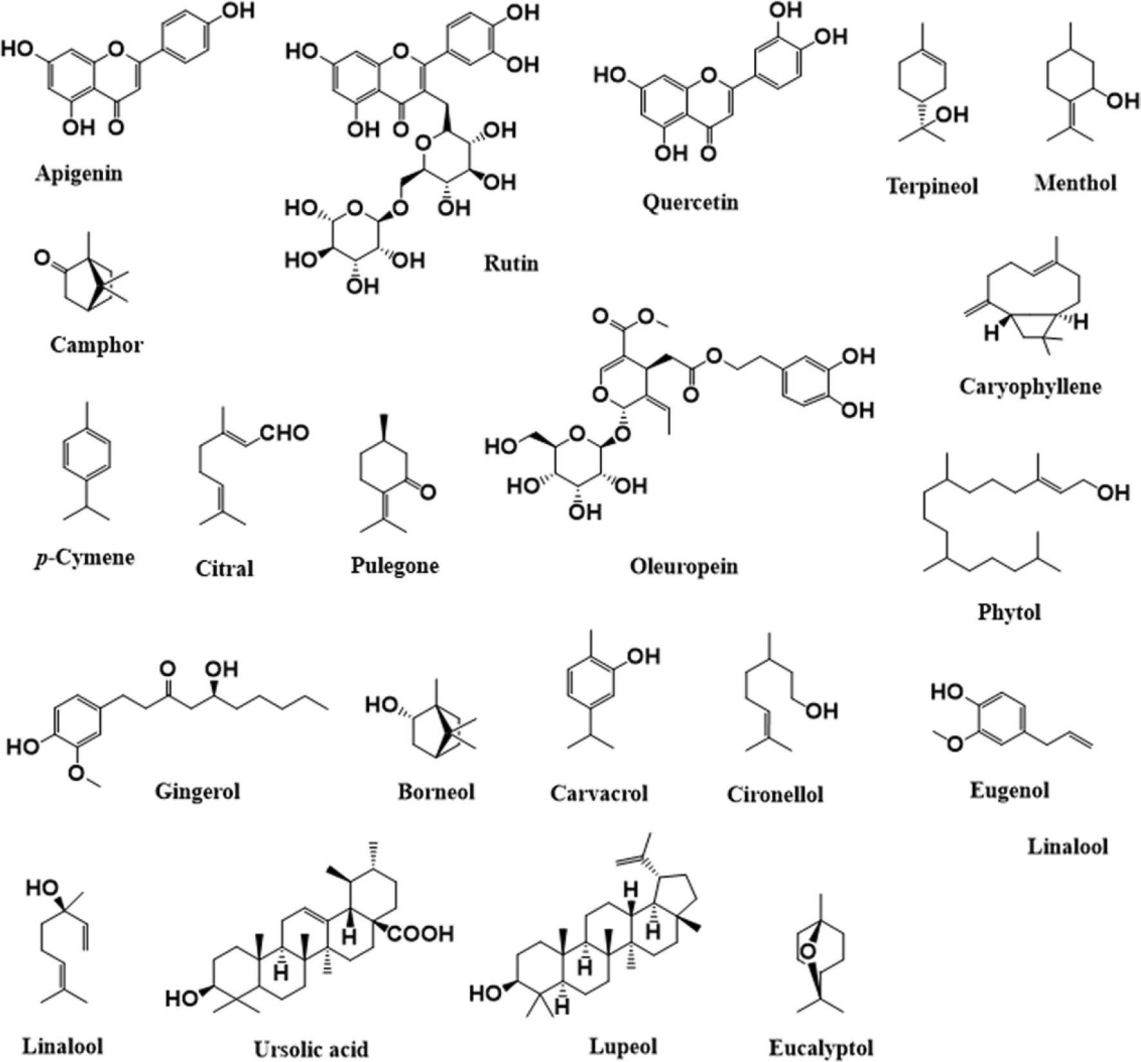
Bioactive compounds isolated from plants with anticonvulsant/antiepileptic and convulsive activities

Flavonoids, often synthesized by the phenylpropanoid pathway, belong to a class of phenolic compounds with a benzo-*γ*-pyrone structure that is ubiquitously distributed in plants [265, 266]. They are the first class of phytochemicals involved in the suppression of seizures in different animal models. Apigenin (Fig. 2) is one of the most common flavones found in *Ajuga integrifolia, Balanites aegyptica, Nicotiana tabacum,* and *Olea europaea* among others. It elicited pronounced anticonvulsant activity in PTC-induced seizures in SD rats as well as KA-induced seizure model through activation of GABA_A_ receptor and inhibition of glutamatergic neurotransmission. Moreover, apigenin possesses inhibitory activity against hydroxyl radical generation through upregulation of reduced glutathione (GSH), consequently, can inhibit neuronal damage in the hippocampal caused by oxidative glutamate toxicity (involved in neuronal death due to epilepsy) [267]. Rutin is a flavonoid glycoside and a constituent of *Balanites aegyptica, Buddleja polystachya, Carissa edulis, Opuntia ficus-indica, Ruta chalepensis* among others with profound in vivo antiepileptic activities. Rutin ameliorated PTZ-kindling in KA-induced seizure upon intraperitoneal (IP) administration. It was devoid of significant anticonvulsant activity against PTZ and MES-induced seizure models (at 800 mg/kg) when administered through the IP route. However, intracerebroventricular administration of rutin suppressed clonic and GTCS in the PTZ-induced model. Thus, the effect on GABA, the glutamate pathway, acetylcholine, glycine, serotonin, and adenosine receptors might be implicated for the observed anticonvulsant activity of rutin. Moreover, the antioxidant activity of rutin may also play a crucial role in its antiepileptic outcome [268]. Quercetin is a flavonoid found in *Ajuga integrifolia, Allium sativum, Balanites aegyptica, Nicotiana tabacum, Olea europaea, Opuntia ficus-indica, Ruta chalepensis,* and *Xanthium stramonium* that exhibited noticeable anticonvulsant activities in different seizure models. In the KA-induced seizure model involving BALB/c mice, quercetin recorded lower seizure scores as compared to the negative control group [269]. It also elicited significant anticonvulsant outcomes after 30 and 60 min of administration in psychomotor seizures induced by 6-Hz simulation. In addition, it also prolonged the onset of seizures and reduced the generalized seizure duration in PTZ-induced convulsions in the male Albino rat at a dose of 10 mg/kg. Furthermore, at 20 mg/kg, quercetin amplified the latency of PIC-induced seizures [6].

### Terpenoids with anticonvulsant activities

#### Monoterpenes

Terpenoids, also known as terpenes or isoprenoids, are naturally occurring compounds derived from isoprene units and predominantly found in all classes of living organisms [270]. Terpenoids are often classified based on the number of carbon atoms or isoprene units (IPU) they possess: monoterpenes (C10, 2 IPU), sesquiterpenes (C15, 3 IPU), diterpenes (C20, 4 IPU), triterpenes (C30, 6 ITU), etc. [271]. Terpenoids in general and monoterpenes specifically are used for the management of CNS disorders including epilepsy. α-Terpineol is monoterpene alcohol obtained from *Artemisia afra, Buddleja polystachya, Croton macrostachyus, Ruta chalepensis,* and *Zingiber officinale*. It has shown significant anticonvulsant activity in PTZ and MES-induced seizure models. Albeit, the exact seizure suppression mechanism of α-terpineol is not known yet [268]. Menthol is a monoterpene found in *Ruta chalepensis* shown to have profound anticonvulsant effects in different animal models. It elicited its antiseizure activity by delaying the onset of clonic and tonic seizures against PTZ-induced convulsions. Moreover, it also suppressed seizures in hippocampal kindled rats. GABA_A_ receptor activation in the hippocampal neurons and thereby inhibition of neuronal excitation (tonic GABAergic inhibition) is believed for the beneficiary effect of menthol against epileptiform [170]. Camphor is monoterpene predominantly found in PMs such as *Artemisia afra, Buddleja polystachya, Ruta chalepensis,* and *Zingiber officinale* among others showed significant anticonvulsant activity in different models. Moreover, it served as a pharmacophore for the synthesis of different anticonvulsant agents. In this regard, benzylidene camphor derivatives containing hydrazone, semicarbazones and thiosemicarbazones exhibited significant antiepileptic activity against MES-induced seizures at 30 mg/kg (comparable to phenytoin) with low neurotoxicity [272]. *p*-cymene is a constituent of *Artemisia afra, Croton macrostachyus, Xanthium stramonium* and *Zingiber officinale* possess anticonvulsant activities. It suppressed convulsions induced by PTZ and MES in mice through modulation of GABAergic neurotransmission via GABA_A_ receptor [273, 274]. Citral is another monoterpene found in *Zingiber officinale* with biological importance for the treatment of CNS malfunction such as epilepsy. It increased the latency time in PTZ-induced seizure in zebrafish larvae model. Its effect is compromised in flumazenil (FMZ) pretreated groups suggesting the contribution of GABA_A_ receptors. Moreover, downregulation of malondialdehyde (MDA)/NO and upregulation of reduced GSH/catalase (CAT) in brain of citral treated groups reiterated its neuroprotective effect [275]. Pulegone is another monoterpene found in *Ruta chalepensis* that significantly increased the latency of convulsions in PTZ-induced seizure models [276]. Oleuropein is a glycosylated Seco-iridoids that can be predominantly found in *Olea europaea* [256]. It unveiled substantial anticonvulsant activity against PTZ-induced seizure through avoidance of neuronal damage via attenuation of generation of reactive oxygen species (ROS) in the epileptic brain [161].

#### Sesquiterpenes and diterpenes

Sesquiterpenes are the other class of terpenoids with potential anticonvulsant activities. β-caryophyllene is a natural sesquiterpene obtained from *Artemisia afra, Croton macrostachyus, Xanthium stramonium,* and *Zingiber officinale*. Contrary to its outcome in PTZ-induced convulsions, *β*-caryophyllene has reduced seizure severity and OS in the KA-induced seizure model. The result revealed the potential of *β*-caryophyllene to suppress seizure by inhibiting thiobarbituric acid reactive species and elevating non-protein thiol levels in the KA model [277]. Diterpenes and their derivatives are among the single compounds that demonstrated relevant antiseizure activities in animal models. Phytol is a component of *Artemisia afra, Buddleja polystachya, Jatropha curcas, *etc. It reduced SE and PLC-induced convulsions by targeting neurotransmitters other than the GABAergic system [268]. 6-GIN, major constituent of *Zingiber officinale* rhizome, is a diterpenoid with potent anticonvulsant activity. It exerted dose-dependent antiepileptic activity against PTZ-induced hyperlocomotion seizure in the zebrafish larvae model. Its anticonvulsant activity is partly associated with the restoration balance between GABA & GLU neurotransmission in the epileptic brain [168].

#### Triterpenes

Triterpenoids are a diverse class of phytochemicals with potential CNS effects such as memory enhancement, ameliorating of depression, suppression of epilepsy, etc. Borneol is a triterpenoid found in *Artemisia afra, Ruta chalepnesis, Xanthium stramonium,* and *Zingiber officinale* with the ability to alleviate ES in different animal models. It produced an enhanced time of onset of clonic seizures in PTZ-kindled mice. Moreover, the PTZ-kindling was counteracted by borneol as manifested by the decrease in lipid peroxidation (LPO) levels, increased superoxide dismutase (SOD), GSH, CAT levels [278]. Carvacrol, a triterpenoid found in *Ruta chalepnesis*, suppressed the onset of clonic seizure in the same model at relatively higher doses. These phytoconstituents showed antiepileptic activities after deactivation of GABA_A_ receptor by FMZ, suggesting the involvement of GABAergic neurotransmission in containing seizures through indirect activation of BZP site of GABA_A_-BZP receptors [279]. Citronellol is also another class of triterpenoid found in different MPs including *Zingiber officinale*. Inhibition of neuronal excitability through voltage-dependent Na^+^ channels is the proposed mechanism for the antiepileptic activity of citronellol. Moreover, it also activates the GABA_A_ receptor and thereby foster GABA neurotransmission in the rat brain [280]. Eugenol is a triterpenoid obtained from *Artemisia afra*. At 100 mg/kg, eugenol suppressed SE and related mortality in PLC-induced SD rats. The involvement of voltage-gated Na^+^ channel in the anticonvulsant activity of eugenol was proved by its weakened effect upon pre-administration of the Na^+^ channel antagonist, riluzole [281]. Linalool is found in *Croton macrostachyus, Ruta chalepensis* and *Zingiber officinale*. It suppressed quinolic acid (QA)-induced seizure (via NMDA antagonism), delayed NMDA-induced convulsions, increase latency onset and duration of clonic seizures in the PTZ-kindling model. The later seizure model also proved the involvement of a wide array of mechanisms despite glutamate blockage [268]. Ursolic acid is a pentacyclic triterpenoid obtained found in *Buddleja polystachya*. It has a profound anticonvulsant activity possibly by modulating the non-BZP sites of the GABA_A_ receptor. In addition, it also showed an anticonvulsant effect in MES- and 6 Hz-induced seizure models through activation of the GABAergic pathway [282]. Lupeol is a triterpenoid found in *Carissa edulis, Croton macrostachyus, Jatropha curcas, Maytenus heterophylla, Nicotiana tabacum, Sida rhombifolia, Xanthium stramonium, *etc. It has shown anticonvulsant activities against PTZ and MES-induced seizure models. Lupeol has increased the mean onset of myoclonic jerks/spasms and differentially protected the mice against mortality [172].

### Proconvulsive phytoconstituents of medicinal plants

At this point, it is worthy to mention that some phytoconstituents have convulsive activity (vigorous jerking of the body and loss of consciousness). Crude extracts or essential oils of some MPs can induce seizure upon systemic or topical administration. Phytoconstituents such as eucalyptol and camphor have shown a significant convulsive effect [283]. For instance, one teaspoon of camphor oil taken orally (by a 3 year child) induced GTCS and respiratory depression within 20 min. On the other hand, eucalyptol induced convulsions characterized by the development of long-term SE and showed developmental delay for at least four years following the event [284]. Thus, attention should be given to antiepileptic MPs which contain camphor (*Artemisia afra, Buddleja polystachya, Ruta chalepensis,* and *Zingiber officinale*) and eucalyptol (*Artemisia afra*) when used by THs to manage the convulsive effect and long-term side-effects. Extensive research could be conducted to determine the tolerable dose which can delimit the protective and convulsive outcomes of camphor and eucalyptol. Overall, the anticonvulsant activities of phytoconstituents included in Table 5 signifies the therapeutic potential of the antiepileptic MPs and the importance of evidence-based phytochemical screening to maximize the benefit of MPs and bring about new AEDs of plant origin.

## Conclusion

Plants have a central role in the traditional medicinal folklore of Ethiopia. Around 96 PS which belong to 43 families were reported for the treatment of epilepsy and related symptoms in different parts of Ethiopia. A portion of these PS was also used for the same purpose in Africa, the Middle East, Asia, and Latin America. The pharmacological activities of nearly one-third of the MPs claimed by the THs for attenuation of seizure in Ethiopia and other parts of the globe were verified by in vivo experiments using different animal and seizure models. The experimentally proved anticonvulsant activities of MPs have presented the importance of indigenous knowledge and the existing traditional healthcare system in the management of epilepsy in different countries, especially in Ethiopia. A strong association between traditional herbal formulations and pharmacological activities of antiepileptic MPs has been established. Yet, the vast majority of the MPs documented in the present review were not screened for their anticonvulsant activities. In addition, the in vivo experiments conducted elsewhere on the target MPs are shallow and not insightful as far as the MOA of crude extracts, solvent fractions, and EOs are concerned. Furthermore, the in vivo pharmacological experiments (anticonvulsant activities) were not accompanied by isolation and characterization of bioactive phytoconstituents responsible for the antiepileptic MPs. Overall, the majority of the PS documented in this review require additional investigation on pharmacological activities, potential targets and mechanism of seizure attenuation, isolation and characterization of bioactive compounds, and toxicological analysis to validate the significance of MPs to tackle epilepsy-associated comorbidities and mortalities.

## Data Availability

Not applicable.

## Abbreviations

AMP: Aminophylline
AEDs: Antiepileptic drugs
AEMNAs: Antiepileptic medications non-adherences
AQ: Aqueous
BIC: Bicuculline
BU: Butanol
CAT: Catalase
CNS: Central nervous system
CH: Chloroform
CAMs: Complementary and alternative medicines
ET: Ethanolic
ES: Epileptic seizures
FMZ: Flumazenil
GTCS: Generalized tonic clonic seizures
GLU: Glutamine
GSK-3: Glycogen synthase kinase-3
HET: Hydroalcoholic/hydroethanolic
HME: Hydromethanolic
IP: Intraperitoneal
INH: Isonicotinic hydrazide acid
IPU: Isoprene units
KA: Kainic acid
LPO: Lipid peroxidation
LMICs: Low- and middle-income countries
MDA: Malondialdehyde
MES: Maximal electroshock
MOA: Mechanism of action
MPs: Medicinal plants
ME: Methanolic
NCAM: National center for complementary and alternative medicines
NMDA: N-methyl-D-aspartate
OS: Oxidative stress
PE: Petroleum ether
PIC: Picrotoxin
PLC: Pilocarpine
PS: Plant species
PTZ: Pentylenetetrazol
QOL: Quality of life
QA: Quinolic acid
ROS: Reactive oxygen species
GSH: Reduced glutathione
SNNP: Southern nations nationalities and peoples
SE: Status epilepticus
STR: Strychnine
SOD: Superoxide dismutase
THS: Traditional healers
FDA: United States Food and Drug Administration
TM: Traditional medicine
VPA: Valproic acid
WHO: World Health Organization and GABA, γ-aminobutyric acid
